# Rho family small GTPase Rif regulates Wnt5a-Ror1-Dvl2 signaling and promotes lung adenocarcinoma progression

**DOI:** 10.1016/j.jbc.2023.105248

**Published:** 2023-09-12

**Authors:** Michiru Nishita, Koki Kamizaki, Kyoka Hoshi, Kana Aruga, Ikumi Nishikaku, Hiroshi Shibuya, Kunio Matsumoto, Yasuhiro Minami

**Affiliations:** 1 Department of Biochemistry, Fukushima Medical University School of Medicine, Fukushima, Japan; 2 Division of Cell Physiology, Department of Physiology and Cell Biology, Kobe University, Graduate School of Medicine, Kobe, Japan; 3 Department of Molecular Cell Biology, Medical Research Institute, Tokyo Medical and Dental University (TMDU), Bunkyo-ku, Tokyo, Japan; 4 Division of Tumor Dynamics and Regulation, Cancer Research Institute, Kanazawa University, Kakuma, Kanazawa, Japan; 5 WPI-Nano Life Science Institute, Kanazawa University, Kakuma, Kanazawa, Japan

**Keywords:** cell invasion, cell polarity, cell proliferation, filopodia, lung cancer, Rho GTPase, Wnt signaling

## Abstract

Rho in filopodia (Rif), a member of the Rho family of small GTPases, induces filopodia formation primarily on the dorsal surface of cells; however, its function remains largely unclear. Here, we show that Rif interacts with Ror1, a receptor for Wnt5a that can also induce dorsal filopodia. Our immunohistochemical analysis revealed a high frequency of coexpression of Ror1 and Rif in lung adenocarcinoma. Lung adenocarcinoma cells cultured on Matrigel established front–rear polarity with massive filopodia on their front surfaces, where Ror1 and Rif were accumulated. Suppression of Ror1 or Rif expression inhibited cell proliferation, survival, and invasion, accompanied by the loss of filopodia and cell polarity *in vitro*, and prevented tumor growth *in vivo*. Furthermore, we found that Rif was required to activate Wnt5a-Ror1 signaling at the cell surface leading to phosphorylation of the Wnt signaling pathway hub protein Dvl2, which was further promoted by culturing the cells on Matrigel. Our findings reveal a novel function of Rif in mediating Wnt5a-Ror1-Dvl2 signaling, which is associated with the formation of polarized filopodia on 3D matrices in lung adenocarcinoma cells.

Filopodia are actin-based, finger-like protrusions that extend from the cell surface. They are dynamic structures that sense chemical and mechanical cues in the environment surrounding the cells (1, 2). Filopodia grow or shrink in response to signals from the extracellular environment, allowing the cell to drive polarized migration (3). Metastatic cancer cells extend filopodium-like protrusions to interact with extracellular matrix (ECM) components around the cells, triggering signaling for cancer cell survival and proliferation (4). Cytonemes are specialized filopodia that can transport signaling proteins between cells (5). Thus, filopodia are used by cells to perform a wide range of functions, and abnormalities in filopodia formation have been associated with various diseases and conditions, including cancer, neurological disorders, and developmental defects (6, 7). Multiple signaling pathways contribute to filopodia formation, and one of the most studied pathways involves the Rho family of small GTPase Cdc42. Cdc42 is activated by guanine nucleotide exchange factors, which promote the exchange of GDP with GTP. Activated Cdc42 binds to and activates downstream effectors, including N-WASP and IRSp53, to induce filopodia formation (8, 9). Another small GTPase, Rho in filopodia (Rif), also called RhoF, induces filopodia formation when overexpressed (10). Different from Cdc42, Rif is a fast-cycling atypical small GTPase that exists in a constitutively active, GTP-bound form in cells (11). It induces filopodia on the peripheral and dorsal cell surfaces through its effector mDia2, whereas Cdc42 primarily induces peripheral filopodia (12). Rif also interacts with IRTKS, an I-BAR family protein closely related to IRSp53, to generate dorsal filopodia (13). However, the roles of Rif-induced filopodia under physiological and pathological conditions remain unclear.

The Wnt family of secreted lipid-modified glycoproteins play essential roles in embryonic development, tissue homeostasis, and diseases (14, 15, 16). They can activate several signaling pathways, including Wnt/β-catenin and Wnt/planar cell polarity (PCP) pathways, by binding to the different receptors, including the Frizzled (Fzd) family of seven-pass transmembrane proteins, at the plasma membrane (17, 18, 19). The Fzd proteins serve as receptors for Wnt ligands and interact with a diverse set of effectors (20, 21, 22), among which the phosphoprotein, Dishevelled (Dvl) protein, acts as a hub for different Wnt signaling pathways (23). Wnt stimulation induces dynamic conformational changes in the Fzd–Dvl complex, which seems to be crucial for defining and initiating distinct intracellular signaling pathways (24, 25). The Ror family receptor tyrosine kinases Ror1 and Ror2 act as receptors or coreceptors for Wnt5a to mediate PCP signaling, which involves the phosphorylation of Dvl (26, 27, 28, 29, 30, 31). Ror1 or Ror2 overexpression induces filopodia formation in numerous experimental systems (32, 33, 34). Moreover, Ror2-mediated Wnt/PCP signaling induces the formation of cytonemes, which mediate the transport of Wnt8a to neighboring cells, thereby triggering Wnt/β-catenin signaling in Wnt-receiving cells (5). Although Ror1 and Ror2 play important roles during embryonic development, their upregulated expression contributes to cancer progression by regulating the proliferation, migration, invasion, survival, and chemoresistance of cancer cells (35). However, whether Ror-induced filopodia formation is associated with tumor progression and the underlying mechanisms remain unclear. Thus, this study aimed to fill this research gap. Here, we uncovered a novel function of Rif in mediating Wnt5a-Ror1-Dvl2 signaling, which is associated with the formation of polarized filopodia on 3D matrices in lung adenocarcinoma (LUAD) cells.

## Results

### Ectopic expression of Ror or Rif induces filopodia formation primarily on the dorsal surface

**Figure 1:**
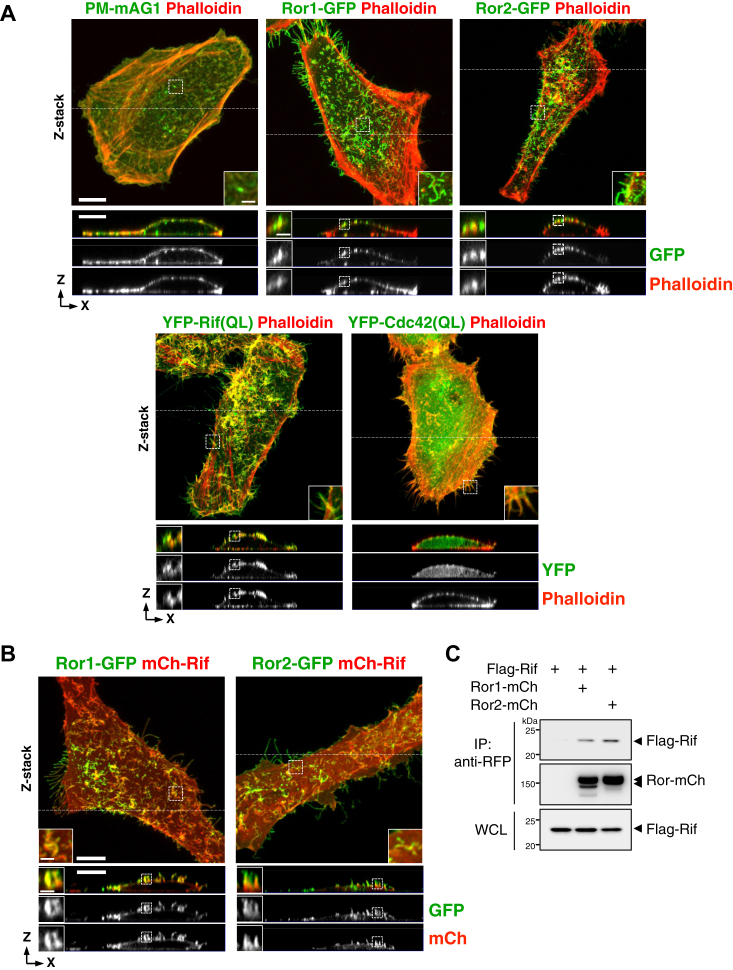
**Ror and Rif induce formation of dorsal filopodia and interact with each other.***A* and *B,* representative images of HeLaS3 cells expressing the indicated fluorescence proteins on a 2D surface. Phalloidin staining was performed to visualize F-actin (*A*). Serial optical confocal z sections are stacked (z-stack), and the xz images sectioned along the *white dotted line* in the z-stack images are shown. Insets show magnified images of *boxed regions*. Images are representative of at least three independent experiments. The scale bars represent 10 μm (main images) and 2 μm (magnified images). *C,* coimmunoprecipitation assay showing the association of Flag-Rif with Ror1-mCherry (mCh) and Ror2-mCh in 293T cells. Whole-cell lysates (WCL) from 293T cells expressing the indicated proteins were subjected to immunoprecipitation (IP) with anti-RFP antibody (that recognizes mCherry), followed by Western blotting. Blots are representative of three independent experiments. Rif, Rho in filopodia

In HeLaS3 cells grown on coverslips, ectopic expression of either Ror1-GFP or Ror2-GFP induced the robust formation of dorsal filopodia, where Ror1 and Ror2 were highly localized (Fig. 1*A*). By contrast, control HeLaS3 cells expressing plasma membrane–targeted monomeric Azami-Green 1 possessed few dorsal filopodia (Fig. 1*A*). The Rho family of small GTPases Rif and Cdc42 can primarily induce the formation of dorsal and peripheral filopodia, respectively (12). Indeed, expression of constitutively active Rif(QL) induced filopodia formation preferentially on the dorsal surface and was localized to the filopodia, whereas Cdc42(QL) induced peripheral filopodia (Fig. 1*A*). Considering the observed similarities between Ror and Rif in filopodia formation and intracellular localization, we determined whether they interact physically and/or functionally. We confirmed that mCherry-Rif and Ror1-GFP or Ror2-GFP colocalized on the dorsal filopodia when they were coexpressed in HeLaS3 cells (Fig. 1*B*). Furthermore, Flag-Rif could be coimmunoprecipitated with either Ror1-mCherry or Ror2-mCherry in 293T cells (Fig. 1*C*), suggesting that Ror and Rif can interact with each other in filopodia.

### Increased *Rif* expression is associated with poor prognosis in patients with LUAD and its aggressiveness

**Figure 2:**
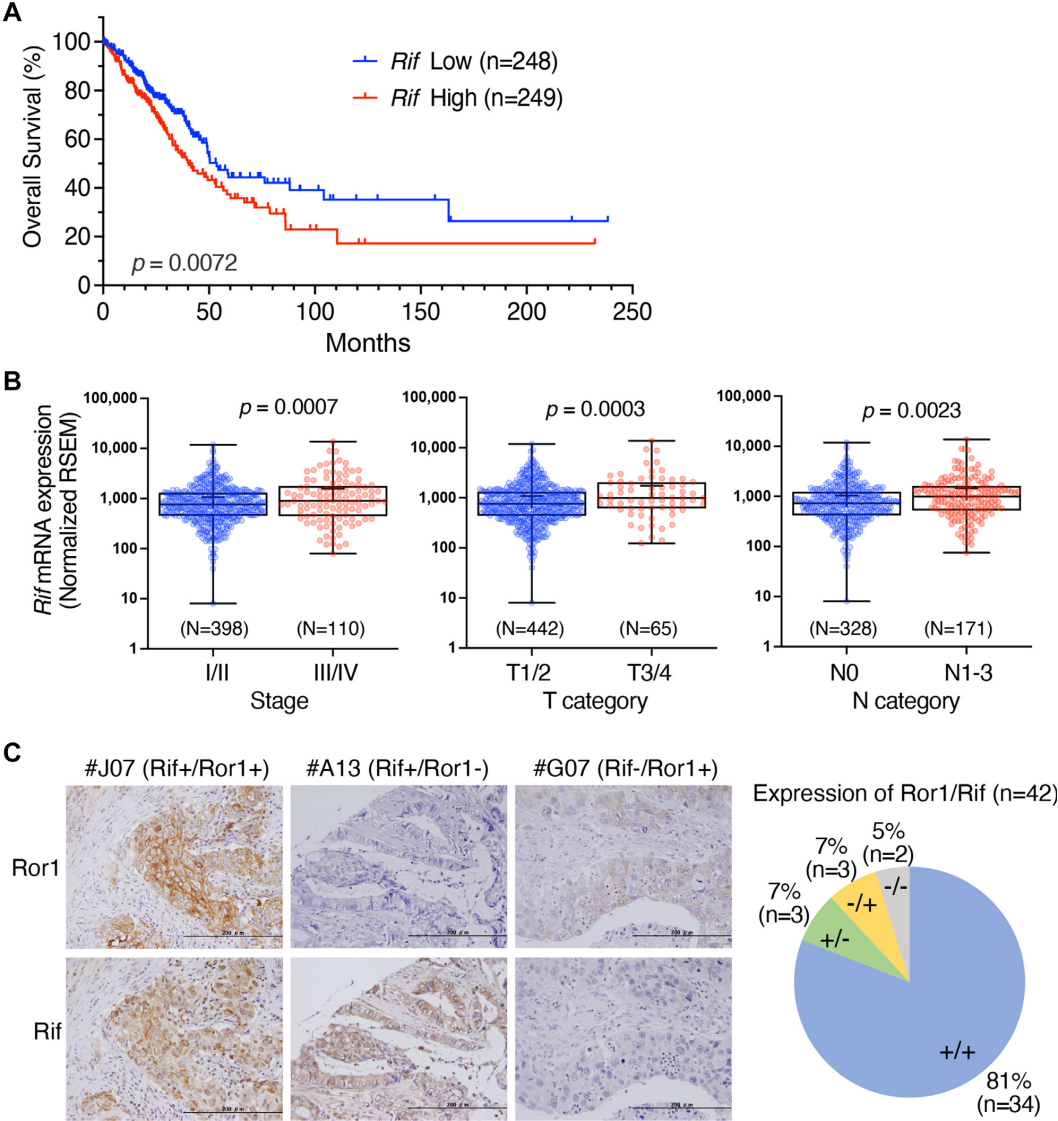
**Higher level of *Rif* expression is associated with poorer prognosis in patients with LUAD and its aggressiveness.***A,* Kaplan–Meier survival analysis of *Rif* high and low expression groups in patients with LUAD. *p* values are obtained from the log-rank test. *B,* expression levels of *Rif* in LUAD were analyzed in correlation with the indicated classified tumor grades. Statistical significance was analyzed using *t* test. *C,* immunohistochemical analysis of Ror1 and Rif on tissue microarray of LUAD. Representative images of double-positive (Rif+/Ror1+) and single-positive (Rif+/Ror1-or Rif-/Ror1+) specimens and a summary of the analyses (n = 42) are shown. The scale bars represent 200 μm. LUAD, lung adenocarcinoma; Rif, Rho in filopodia.

Although Ror1 has been implicated in the progression of various types of cancer, only a few reports have suggested an involvement between Rif and cancer progression (36, 37, 38). Therefore, we explored cancer types showing a correlation between the expression of *Rif* mRNA and prognosis of patients using OncoLnc (www.oncolnc.org), a website tool that links the The Cancer Genome Atlas (TCGA) survival data to mRNA expression levels. Results revealed that pancreatic adenocarcinoma and acute myeloid leukemia showed the highest and second highest correlation, respectively, followed by LUAD. We focused on LUAD because no report has suggested a role for Rif in LUAD progression, whereas Ror1 has been implicated in LUAD progression (39, 40). We extracted the data of *Rif* expression, together with the clinical features and survival information of patients with LUAD from the TCGA data portal. As shown in Figure 2*A*, patients with higher *Rif* expression exhibited poorer overall survival than those with low *Rif* expression. Furthermore, *Rif* expression was significantly upregulated in advanced-stage cancer (stage III/IV) compared with early-stage cancer (stage I/II) (Fig. 2*B*). Moreover, *Rif* expression was significantly higher in patients with T3/4 and N1-3 than in patients with T1/2 and N0, respectively (Fig. 2*B*). Given the smaller number of patients with M1 (n = 25) compared with patients with M0 (n = 241), we failed to detect a significant difference in *Rif* expression between the two stages (*p* = 0.7895) (data not shown). To examine the protein expression of Ror1 and Rif, we performed immunohistochemistry on LUAD tissue arrays. Among the 42 patients with LUAD, 34 (81%) were positive for both Rif and Ror1 in tumor cells (Fig. 2*C*), indicating that Rif and Ror1 are highly expressed in LUAD tissues.

### Ror1 and Rif are colocalized on the front surface with filopodia of 3D-migrating cells

**Figure 3:**
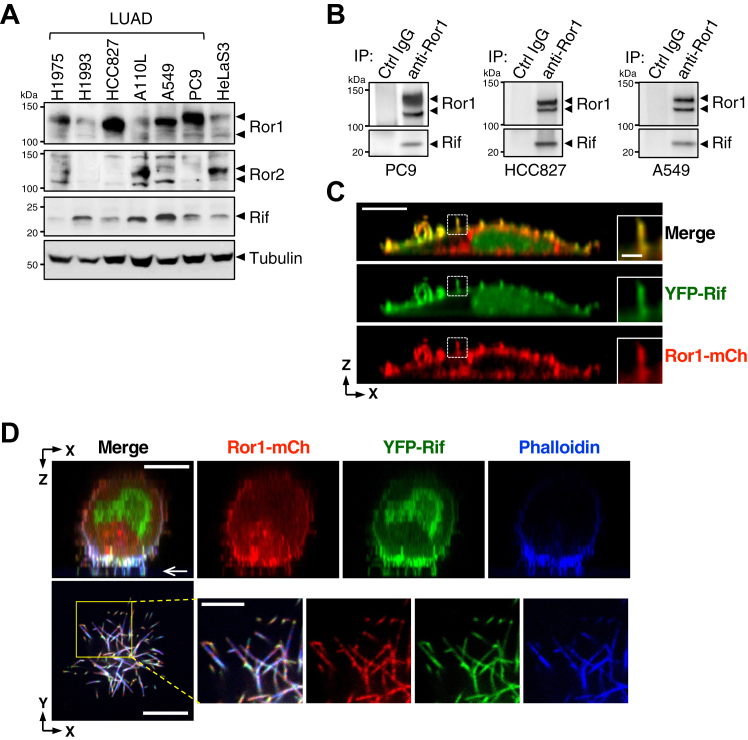
**Ror1 and Rif are colocalized at filopodia in LUAD cells.***A,* Western blot analysis of Ror1, Ror2, and Rif in LUAD cell lines, showing expression of both Ror1 and Rif were detectable in all LUAD cell lines examined. Blots are representative of two independent experiments. *B,* coimmunoprecipitation assay, showing association between Rif and Ror1 at endogenous protein levels in the indicated LUAD cells. Whole-cell lysates were subjected to immunoprecipitation (IP) with anti-Ror1 antibody or control immunoglobulin G (isotype matched), followed by Western blotting. Blots are representative of six (PC9), three (HCC827), and two (A549) independent experiments. *C* and *D,* representative xz-images of PC9 cells expressing YFP-Rif and Ror1-mCh on a 2D surface (*C*) or Matrigel (*D*), showing colocalization of these proteins at filopodia. Phalloidin staining in (*D*) shows highly accumulated F-actin on the front side containing filopodia. In (*D*), the xy images (*lower panels*) sectioned along the *white arrow* in xz confocal image (*upper panels*) are shown. Images are representative of at least three independent experiments. The scale bars represent 10 μm. Magnified images of *boxed regions* are shown on the *right*. The scale bars in magnified images represent 2 μm (*C*) and 5 μm (*D*). LUAD, lung adenocarcinoma; Rif, Rho in filopodia.

Coexpression of Rif and Ror1 was confirmed in panels of LUAD cell lines through Western blotting (Fig. 3*A*). Coimmunoprecipitation analyses confirmed the physical interaction between endogenous Ror1 and Rif in PC9, HCC827, and A549 cells (Fig. 3*B*). Similar to HeLaS3 cells, PC9 and HCC827 cells, expressing Ror1-mCherry and YFP-Rif, formed filopodia on their dorsal surfaces when cultured on 2D coverslips, and both Ror1 and Rif are colocalized at the filopodia (Figs. 3*C* and S1*A*). We next cultured these cells on a bed of Matrigel, a basement membrane–like ECM, which provides cells with a more physiological 3D microenvironment. Interestingly, the cells robustly developed F-actin–rich filopodia on their surface facing the Matrigel (Figs. 3*D* and S1*B*), a characteristic observed on the front surface of migrating cells. Furthermore, Ror1-mCherry and YFP-Rif accumulated on the front surface with filopodia (Figs. 3*D* and S1*B*), suggesting that Ror1 and Rif are involved in the formation and/or function of filopodia during cancer cell migration through 3D matrices. YFP-Rif was also detectable in the nucleus of PC9 cells regardless of the culture conditions (Fig. 3, *C* and *D*), although its physiological relevance is presently unclear.

### Ror1 and Rif promote proliferation, survival, and invasion of LUAD cells *in vitro* and tumor development *in vivo*

**Figure 4:**
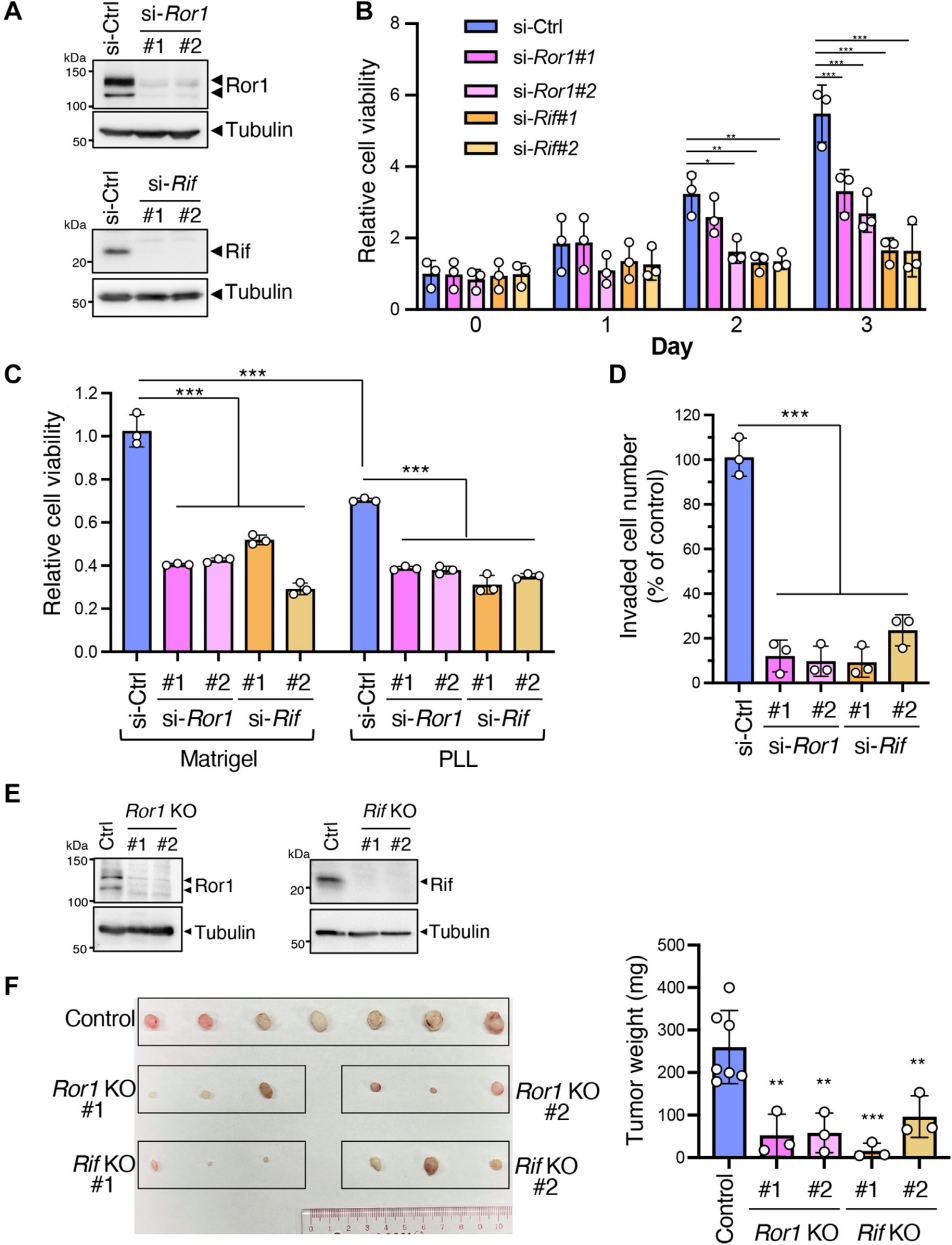
**Ror1 and Rif promote cell proliferation, survival, and invasion *in vitro* and tumor development *in vivo*.***A,* Western blot analysis showing efficient knockdown of *Ror1* and *Rif* by the respective siRNAs in PC9 cells. Images are representative of at least three independent experiments. *B* and *C,* effects of knockdown of *Ror1* or *Rif* on cell proliferation (*B*) and survival (*C*). Viability of PC9 cells transfected with the indicated siRNAs were assessed in media containing 10% (*B*) or 1% (*C*) FBS by using the WST-8 assay, as described in Materials and methods. In (*C*), cells were cultured on either a PLL-coated 2D surface or Matrigel for 3 days before measuring cell viability. Data are expressed as mean ± SD of three independent experiments, each performed in triplicate (*B*) or mean ± SD of three technical replicates of a representative experiment out of three independent experiments. ∗*p* < 0.05, ∗∗*p* < 0.01, ∗∗∗*p* < 0.001, Tukey’s test. *D,* Transwell invasion assay showing decreased invasion of PC9 cells treated with si-*Ror1* or si-*Rif*. Data are expressed as mean ± SD of three independent experiments. ∗∗∗*p* < 0.001, Dunnett’s test. *E,* Western blot analysis showing ablated expression of Ror1 and Rif in the respective KO PC9 cells. Blots are representative of two independent experiments, respectively. *F,* representative images of tumor tissues generated by subcutaneous transplantation of control, *Ror1*-KO, or *Rif*-KO PC9 cells into nude mice. Graph shows mean weight of tumor tissues. Data are expressed as mean ± SD of seven (control) and three (*Ror1* KO#1, #2, and *Rif* KO#1, #2) xenografts from three and seven mice, respectively. ∗∗ *p* < 0.01, ∗∗∗ *p* < 0.001, Dunnett’s test. PLL, poly-L-lysine; Rif, Rho in filopodia.

Ror1 promotes the proliferation and survival of LUAD cells (39). PC9 cells were treated with siRNAs against either *Ror1* or *Rif* (Fig. 4*A*), and their effects on cell viability were assessed using the WST-8 assay. Under normal culture conditions with 10% fetal bovine serum (FBS), knockdown of either *Ror1* or *Rif* significantly reduced cell proliferation when compared with control cells (Fig. 4*B*). Thus, similar to Ror1, Rif was required for PC9 cell proliferation. We next cultured the cells in media containing 1% FBS for 3 days on culture plates precoated with either poly-L-lysine (PLL), which facilitates cell adhesion, or Matrigel. The viability of control siRNA-transfected cells was significantly higher when cultured on Matrigel than on a PLL-coated surface (Fig. 4*C*). *Rif* or *Ror1* knockdown significantly decreased cell viability regardless of the substrata (Fig. 4*C*). Thus, Matrigel provides cancer cells with a prosurvival microenvironment, and both Ror1 and Rif may mediate this effect by regulating filopodia formation and/or function. We also examined whether Ror1 and Rif are required for invasive migration through Matrigel by using a Transwell invasion assay. siRNA against either *Ror1* or *Rif* drastically inhibited the invasive migration of the cancer cells (Fig. 4*D*). These siRNAs also inhibited the proliferation and invasive migration of HCC827 cells (Fig. S1, *C*–*E*). To study the roles of Ror1 and Rif in tumor development *in vivo*, we generated *Ror1*- or *Rif*-KO PC9 cells by using the CRISPR-Cas9 system (Fig. 4*E*) and injected them subcutaneously into nude mice. The average weight of tumors developed from either *Ror1*- or *Rif*-KO PC9 cells was significantly lower than that developed from control PC9 cells (Fig. 4*F*), indicating that Ror1and Rif play crucial roles in tumor development of PC9 cells *in vivo*.

### Ror1 and Rif are required to establish front–rear polarity and invasive activity on matrigel

**Figure 5:**
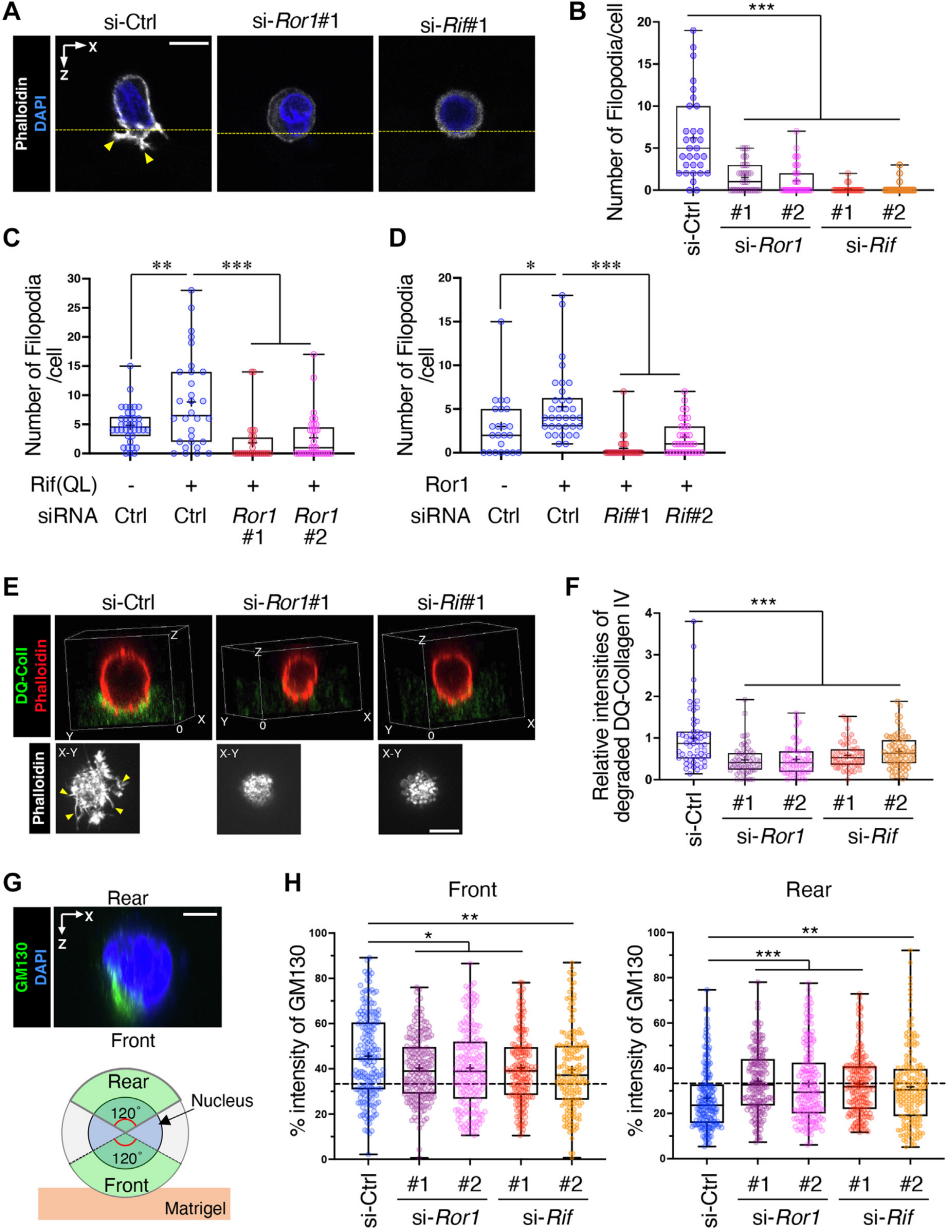
**Ror1 and Rif are required to establish front–rear polarity and invasive activity on Matrigel.***A* and *B,* PC9 cells were cultured on Matrigel for 1 h. Representative xz-images of the cells showing protrusion of filopodia (*yellow arrowheads*) in control cells (*A*). *Yellow dotted lines* indicate the surface of the Matrigel. Images are representative of three independent experiments. Number of filopodia are quantified (*B*). Data are presented as a *box-and-whisker plot*. n = 31 (si-Ctrl, si-*Ror1*#1), 38 (si-*Ror1*#2), 24 (si-*Rif*#1), and 21 (si-*Rif*#2) cells from three independent experiments. ∗∗∗*p* < 0.001, Dunnett’s test. *C* and *D,* filopodia formation induced by ectopic expression of Rif(QL) and Ror1 was suppressed by siRNAs against Ror1 and Rif, respectively. PC9 cells stably expressing Flag-Rif(QL) (*C*) or Ror1-mCherry (*D*), respectively, were transfected with siRNAs against *Ror1* or *Rif* as indicated and assessed for filopodia formation as shown in (*A*). Data are presented as a *box-and-whisker plot*. n = 28 to 38 (*C*) and 23 to 34 cells (*D*) from three independent experiments, respectively. ∗*p* < 0.05, ∗∗*p* < 0.01, ∗∗∗*p* < 0.001, Tukey’s test. *E* and *F,* PC9 cells were cultured on Matrigel containing DQ-collagen IV for 2 h. Representative 3D images (*upper panels*) and xy images sectioned around the *bottom surface* of the cells (*lower panels*) are shown. *Yellow arrowheads* indicate filopodia in control cells. Images are representative of three independent experiments. Intensity of the degraded DQ-collagen IV was quantified (*F*). Data are presented as a *box-and-whisker plot*. n = 57 (si-Ctrl), 60 (si-*Ror1*#1), 55 (si-*Ror1*#2), 59 (si-*Rif*#1), 68 (si-*Rif*#2) cells from three independent experiments. ∗∗∗*p* < 0.001, Dunnett’s test. *G* and *H,* PC9 cells were cultured on Matrigel for 30 min, and the Golgi apparatus (GM130) and nucleus (4′,6-diamidino-2-phenylindole) were stained. Representative xz-image stacked along the *y*-direction are shown (*G*, *upper panel*). Images are representative of three independent experiments. Schematic (*G*, *lower panel*) shows the front and rear of a cell (*green*) defied as the 120° sectors emerging from the center of the nucleus toward the front and rear. Fluorescence intensity of GM130 within the front and rear sectors were quantified (*H*). *Dotted lines* in the graph indicate the level of expected random orientation of 33%. Data are presented as a *box-and-whisker plot*. n = 178 (si-Ctrl, si-*Ror1*#2) and 177 (si-*Ror1*#1, si-*Rif*#1, si-*Rif*#2) cells from three independent experiments. ∗*p* < 0.05, ∗∗*p* < 0.01, ∗∗∗*p* < 0.001, Dunnett’s test. The scale bars represent 10 μm (*A* and *E*) and 5 μm (*G*). DQ, dye-quenched; Rif, Rho in filopodia.

Phalloidin staining revealed that the cells treated with either *Ror1* or *Rif* siRNA lost front–rear polarity and filopodia (Fig. 5*A*). Quantification revealed a significant reduction in the number of filopodia in the *Ror1*- or *Rif*-knockdown cells (Fig. 5*B*). Furthermore, filopodia formation induced by ectopic expression of Rif(QL) and Ror1 was suppressed by siRNAs against Ror1 and Rif, respectively (Fig. 5, *C* and *D*), indicating that Ror1 and Rif are mutually required for filopodia formation. Thus, it is likely that Ror1 and Rif act interdependently rather than independently in regulating filopodia formation. To gain further insights into the association between filopodia formation and invasive activity, we cultured the cells on Matrigel containing dye-quenched (DQ)-collagen IV, which became highly fluorescent upon degradation. Because collagen IV is a major component of Matrigel, fluorescent signals from degraded DQ-collagen IV represent the invasive activity of the cells (41). At 2 h after plating, we detected bright fluorescent signals around the filopodia-rich front surface in control cells (Fig. 5, *E* and *F*). Suppression of *Ror1* or *Rif* expression not only inhibited filopodia formation but also reduced the fluorescence intensity of degraded DQ-collagen IV (Fig. 5, *E* and *F*), indicating that both Ror1 and Rif are required for filopodia formation and collagen IV degradation at the front surface. Notably, reduced degradation of DQ-collagen IV in si-*Rif*#1-transfected cells was reverted by the ectopic expression of WT Rif, but not its GDP-bound mutant (Fig. S2), indicating that Rif promotes collagen IV degradation in a GTP binding––dependent manner. Considering that these processes involve the polarized transport of membranes and proteins, including matrix metalloproteinases (42), we examined whether Ror1 and Rif might affect the orientation of the Golgi apparatus. Cells cultured on Matrigel for 30 min were assessed for their Golgi distribution by measuring the intensity of GM130, a *cis*-Golgi marker, within the 120° sector emerging from the center of the nucleus toward the front and rear (Fig. 5*G*). The mean intensity of GM130 in control cells was substantially higher in the front and lower in the rear than expected for a uniform distribution (33%), indicating a polarized distribution of the Golgi toward the front (Fig. 5, *G* and *H*). In *Ror1* or *Rif* knockdown cells, the mean intensity of GM130 were significantly decreased and increased in the front and rear, respectively, as compared with those in control cells (Fig. 5*H*), indicating that both Ror1 and Rif are required for the efficient reorientation of the Golgi toward the front side, thereby contributing to the establishment of front–rear polarity.

### Rif augments Wnt5a-Ror1-Dvl2 signaling in PC9 cells cultured on matrigel

Rif is a fast-cycling atypical small GTPase that binds GTP predominantly in cells (11). To confirm this, we performed an effector pull-down assay using glutathione-*S*-transferase (GST)-mDia1-Rho-binding domain (RBD) fusion protein, which constitutively coprecipitated the GTP-bound form (QL) but not the GDP-bound form (TN) of Rif expressed in 293T cells (Fig. S3*A*). The levels of coprecipitated Rif were comparable between WT Rif and Rif(QL) (Fig. S3*A*), indicating that WT Rif exists predominantly in the GTP-bound form in the cells. Consistent with this, GTP-bound Rif was highly expressed at the endogenous level in PC9 cells, whereas GTP-bound RhoA was only weakly expressed as assessed by GST-rhotekin-RBD pull-down assay (Fig. S3*B*). Furthermore, the level of GTP-bound Rif remained unaltered after *Ror1* knockdown in PC9 cells (Fig. S3*C*). These results strongly suggest that most, if not all, of Rif expressed in PC9 cells exists in a GTP-bound form irrespective of Ror1 expression.

**Figure 6:**
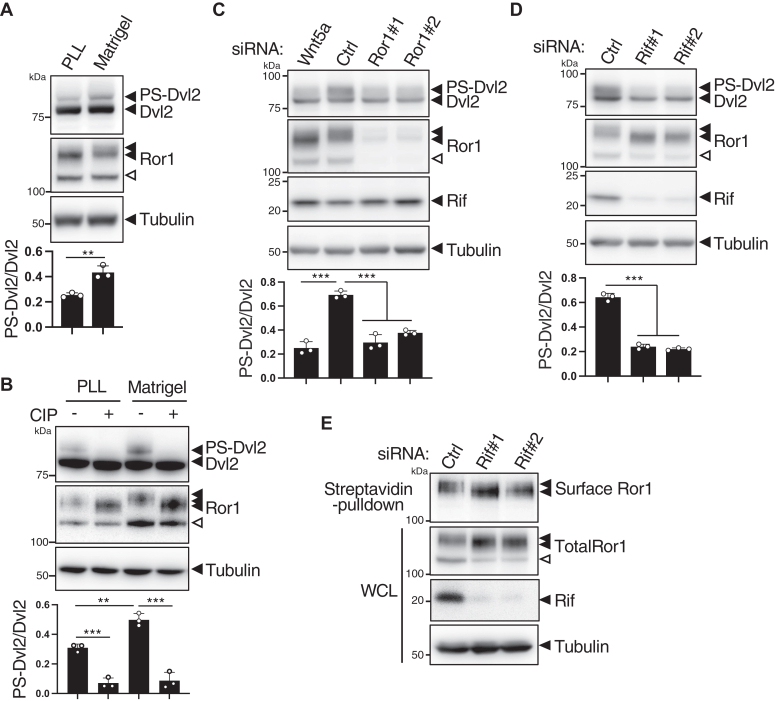
**Rif is required to activate Wnt5a-Ror1-Dvl2 signaling in PC9 cells cultured on Matrigel.***A–D,* untreated PC9 cells (*A* and *B*) or PC9 cells treated with the indicated siRNAs (*C* and *D*) were cultured on either a PLL-coated plate (*A* and *B*) or Matrigel (*A*–*D*). Cells were lysed and analyzed by Western blotting. For dephosphorylation of Dvl2 and Ror1, cell lysates were treated with CIP before subjected to Western blotting (*B*). *Black and white arrowheads* in anti-Ror1 blots represent the 130 and 115 kDa forms of Ror1, respectively. Blots are representative of three independent experiments. Ratios of phosphorylated and shifted Dvl2 (PS-Dvl2) to unshifted Dvl2 (Dvl2) are shown as mean ± SD of three independent experiments. ∗∗*p* < 0.01, ∗∗∗*p* < 0.001, *t* test (*A*), Dunnett’s test (*C* and *D*). *E,* PC9 cells transfected with *Rif* siRNAs were cultured in cell-culture plates and subjected to cell surface biotinylation. Biotin-labeled surface proteins isolated by streptavidin pulldown and whole-cell lysates (WCL) were analyzed by Western blotting. Blots are representative of three independent experiments. CIP, calf intestinal alkaline phosphatase; Dvl, Dishevelled; PLL, poly-L-lysine; PS-Dvl, phosphorylated and shifted Dvl2; Rif, Rho in filopodia.

Ror1 acts as a receptor for Wnt5a to induce the noncanonical Wnt/Dvl2 signaling (26, 27, 43). It has also been reported that Wnt5a is highly expressed in metastatic lung cancer cells (44). We therefore examined whether Wnt5a contributes to high invasiveness of PC9 cells by using *Wnt5a*-KO PC9 cells (Fig. S4*A*). *Wnt5a*-KO PC9 cells showed decreased ability to invade through Matrigel as compared to control PC9 cells (Fig. S4*B*). Furthermore, *Wnt5a*-KO PC9 cells protruded reduced number of filopodia, which could be restored by recombinant Wnt5a treatment (Fig. S4*C*). These results suggest that autocrine Wnt5a production is responsible for the invasive properties of PC9 cells. Similarly, Dvl2 was required for invasive migration of PC9 cells through Matrigel, although it was dispensable for their proliferation (Fig. S5, *A*–*C*), suggesting that Wnt5a-Ror1 signaling regulates cell invasion and proliferation in a Dvl2-denpendent and Dvl2-independent manner, respectively. Thus, we studied the role of Rif in Wnt5a-Ror1-Dvl2 signaling. It has been appreciated that the activation of Wnt5a-Ror1-Dvl2 signaling can be assessed by the phosphorylation-dependent electrophoretic mobility shifts of Ror1 and Dvl2 as reliable surrogate markers (26, 27, 43). Western blotting analysis revealed two major bands of Ror1 at approximately 130 and 115 kDa in PC9 cells. Interestingly, both Ror1 and Dvl2 underwent mobility shifts upon culturing the cells on Matrigel (Fig. 6, *A* and *B*), which was completely reversed by calf intestinal alkaline phosphatase treatment (Fig. 6*B*), indicating that these mobility shifts occur in a phosphorylation dependent manner. We then examined whether Wnt5a is involved in these phosphorylation events. As a result, in PC9 cells cultured on Matrigel, *Wnt5a* knockdown (Fig. S6) inhibited the phosphorylation of both Ror1 and Dvl2 (Fig. 6*C*). Furthermore, *Ror1* knockdown inhibited the phosphorylation of Dvl2 (Fig. 6*C*), indicating that Wnt5a–Ror1–Dvl2 signaling pathway is activated in a cell-autonomous manner. Finally, we examined whether Rif is involved in this signaling pathway. Intriguingly, *Rif* knockdown substantially reduced the phosphorylation of both Ror1 and Dvl2 in PC9 cells cultured on Matrigel (Fig. 6*D*), suggesting that Rif might be a critical regulator acting in Wnt5a-Ror1-Dvl2 signaling under these culture conditions. To confirm whether Rif affects Ror1 phosphorylation at the cell surface, we performed surface biotinylation under 2D culture conditions, where Ror1 was phosphorylated to a certain extent (Fig. 6*B*). The 130 kDa, but not the 115 kDa form of Ror1, which undergoes phosphorylation in a manner dependent on Rif, is biotinylated and therefore located at the cell surface (Fig. 6*E*). Taken together, these results suggest that Rif might be required for the cell-autonomous activation of Wnt5a-Ror1-Dvl2 signaling at the cell surface, which would be further promoted by culturing cells on Matrigel.

## Discussion

Unlike Cdc42, Rif can induce filopodia formation on the dorsal surface (10, 12). Importantly, Cdc42-induced filopodia contain vinculin-rich focal complexes at their tips, which play an important role in integrin-mediated cell–ECM attachment (45, 46). By contrast, Rif-induced filopodia lack such structures (10, 45, 46), which is likely favorable for dorsal filopodia to prevent the attachment of their tips to the substratum. With this respect, the unconventional myosin motor, Myo10, has been identified at the filopodia tips and it drives the formation of *dorsal filopodia* that fail to adhere to the substratum (47). Myo10-induced dorsal filopodia regulate axonal outgrowth, cell migration, and tunneling nanotube formation in neuronal cells (48, 49, 50, 51). Thus, it can be envisaged that Rif-induced dorsal filopodia might play their roles by using somewhat similar machinery, which was seen in neurons and other types of cells.

Higher expression of Rif is associated with poorer clinical outcomes and contributes to cell proliferation and invasion in some cancers, including pancreatic adenocarcinoma (38) and hepatocellular carcinoma (36). However, the role of Rif-induced filopodia formation in cancer progression remains largely unclear. In this study, TCGA cohort analysis has revealed higher expression of *Rif* and its association with poorer clinical outcomes in LUAD, while Ror1 is overexpressed and promotes cell proliferation and survival in LUAD (39). Our immunohistochemical analysis confirmed the high frequency of coexpression of Ror1 and Rif at higher levels in LUAD tissues. Similar to HeLaS3 cells, PC9 and HCC827 LUAD cells formed dorsal filopodia when cultured on 2D coverslips. However, these LUAD cells cultured on Matrigel failed to spread on the Matrigel surface, unlike those on the 2D-platic surface, and instead formed actin-rich membrane protrusions containing massive filopodia on the side facing the Matrigel. This led to the generation of front–rear polarity, which is required for cell migration. Thus, the membrane protrusions generated in the PC9 and HCC827 cells on Matrigel were likely equivalent to the pseudopods of migrating cells. Consistent with this hypothesis, melanoma cells migrating in 3D collagen matrices extend filopodia from their pseudopods in a Myo10-dependent manner (52).

The filopodia generated in PC9 and HCC827 cells on Matrigel may function as invadopodia, which are actin-based membrane protrusions that contribute to cancer cell migration through 3D matrices. Microtubules also penetrate into long invadopodia, where the kinesin motor protein KIF1C regulates their further elongation (53, 54). Invadopodia primarily degrade and remodel the surrounding ECM by recruiting various membrane and secretory proteins, including matrix metalloproteinases, in a manner dependent on a polarized distribution of the Golgi (42, 55, 56). In fact, we detected a robust degradation of collagen IV around the cellular front containing filopodia and polarized Golgi distribution toward them. These filopodia may also act independently of proteases for cell migration through Matrigel by mechanically opening channels within the matrices and generating protrusive forces at the front surface, as recently proposed for invadopodia (57).

We found a novel effect of Matrigel culture on Wnt5a-Ror1-Dvl2 signaling in PC9 cells. Ror1 accumulates at the front surface of cells under these culture conditions. Thus, Wnt5a-Ror1-Dvl2 signaling may be locally activated in a cell autonomous manner, which consequently induces reorientation of the Golgi toward the front side of the nucleus. This notion is supported by the fact that Wnt5a-Dvl signaling is constitutively activated in embryonic fibroblasts and required for Golgi reorientation (58). Considering the role of the Golgi in the secretory pathway, the transport of Wnt5a might also be oriented toward the front of the cells, resulting in a feed-forward activation of Wnt5a-Ror1-Dvl2 signaling. As shown in Figure 6*C*, knockdown of either *Wnt5a* or *Ror1* decreased the PS-Dvl2/Dvl2 ratios to almost the same level in the presence of Matrigel, indicating that Ror1-mediated Dvl2 phosphorylation is dependent mostly, if not entirely, on autocrine Wnt5a under our experimental condition. However, we cannot rule out the possibility that Matrigel might contain active Wnts that may affect cellular response independently of Ror1. Our findings also uncovered a novel role for Rif in Wnt5a-Ror1-Dvl2 signaling. *Rif* knockdown reduced Ror1 phosphorylation on the cell surface without affecting its cell surface protein levels. Therefore, Rif might be required for the efficient recognition of Wnt5a by cell surface Ror1. Further studies are warranted to elucidate the mechanisms by which Rif regulates Wnt5a-Ror1-Dvl2 signaling, eventually leading to cellular proliferation, survival, and invasion of LUAD. It is also of interest to investigate the role of Fzd receptors in Wnt5a-Ror1-Dvl2 signaling, since Fzds are known to serve as primary receptors for Wnt-Dvl signaling (20, 21, 22, 24, 25). In fact, Fzd2 and Wnt5a/b (among 10 Fzds and 16 Wnts) are known to be expressed at higher levels in several metastatic cancer cells, including lung cancer cells (44). Further studies will be required to clarify the possible role of Fzds in Wnt5a-Ror1-Dvl2 signaling regulated by Rif in LUAD cells. Since higher expression of *Rif* is associated with poorer clinical outcomes and contributes to both cell proliferation and invasion in LUAD, Rif may serve as a suitable target for the development of novel diagnostic and therapeutic approach toward LUAD.

## Experimental procedures

### Plasmids and siRNAs

*Ror1*, *Ror2*, and *Rif* cDNAs encoding human Ror1, Ror2, and Rif were isolated from HeLaS3 cells through PCR amplification and subcloned into pEGFP, pmCherry, pBabe-puro and pCMV-Flag vectors. cDNA encoding Ror1-mCherry was subcloned into PiggyBac transposon-based vector PB-EF1-MCS-IRES-Neo (SBI, Mountain View, CA, USA). Plasmids encoding YFP-Rif(QL) and -Rif(TN) carrying the Q77L and T33N point mutations, respectively, were generated through site-directed mutagenesis. A plasmid encoding YFP-Cdc42(QL) was provided by M. Endo (Kobe University, Japan). A plasmid encoding plasma membrane–targeted monomeric Azami-Green 1 was purchased from MBL (Tokyo, Japan). The cDNA encoding mouse mDia1 was provided by N. Watanabe (Kyoto University, Japan). A plasmid encoding GST-mDia1-RBD fusion protein was constructed by subcloning the fragment encoding aa 82 to 379 into pGEX vector. A plasmid encoding GST-rhotekin-RBD was described previously (59). The following custom-designed siRNAs were purchased from Sigma-Aldrich (St Louis, MO, USA): si-*Ror1*#1 (5′-CCCAGAAGCUGCGAACUGUUU-3′); si-*Ror1*#2 (5′-CAGCAAUGGAUGGAAUUUCAAUU-3′); si-*Dvl2*#1, (5′-CAUGGAGAAGUACAACUUCUU-3′); and si-*Wnt5a* (60). The following pre-designed siRNAs were purchased from Sigma-Aldrich: si-*Rif1*#1 (SASI_Hs01_00122478); si-*Rif*#2 (SASI_Hs01_00122480); si-*Dvl2#2* (SASI_Hs01_00104204); and negative control (SIC-001). Since si-*Rif1*#1 is directed against the 3′-UTR of *Rif*, it was used together with plasmids encoding *Rif* without its 3′-UTR for rescue experiments.

### Cell culture, transfection, and retroviral infection

Human LUAD cell lines PC9, A110L, H1993, and H1975 were provided by Y. Maniwa (Kobe University), and HCC827 (which express luciferase stably) and A549 cells were purchased from JCRB cell bank. These cells were maintained in RPMI1640 medium (Nacalai Tesque) containing 10% (v/v) FBS. The short-tandem repeat profiles of PC9, HCC827, and A549 cells were analyzed (BEX CO., LTD), and we confirmed that these cells were not contaminated. HeLaS3 and 293T cells were maintained in Dulbecco's modified Eagle's medium (Nacalai Tesque) supplemented with 10% (v/v) FBS. All cells were incubated at 37 °C with 5% CO_2_ and 90% humidity. The cells were transfected with the respective plasmids and siRNAs by using ViaFect (Promega) and Lipofectamine RNAiMAX (Thermo Fisher Scientific), respectively, in accordance with the manufacturer’s instructions. To obtain PC9 cells stably expressing Ror1-mCherry, cells were transfected with PB-EF1-MCS-IRES-Neo vector containing Ror1-mCherry together with Super PiggyBac transposase expression vector (SBI) and selected with 400 μg/ml G418. Recombinant retroviruses were produced with pBabe-puro-Rif (WT, QL, or TN) or its empty vector to infect PC9 cells as described previously (53). Puromycin-resistant cells were collected for analysis.

### Antibodies and reagents

Rabbit anti-Ror2 antibodies were prepared as described previously (61). The following antibodies were obtained commercially: mouse anti-GM130 (35, BD Biosciences), anti-RFP (165-3, MBL), anti-GFP (JL-8, Takara), anti-RhoA (26C4, Santa Cruz), anti-DYKDDDDK (Flag)-tag (1E6, Fujifilm Wako, Osaka, Japan), and anti-β-actin (AC-15, Sigma-Aldrich); rabbit anti-RhoF for Western blotting (12290-1-AP, Proteintech, Rosemont; ab101349, abcam) and immunohistochemistry (ab101349, abcam), anti-Ror1 for Western blotting (#16540, CST) and immunohistochemistry (#4102, CST), anti-Dvl2 (#3216, CST), anti-Wnt5a (ab179824, abcam), and horseradish peroxidase–conjugated anti-α-tubulin (PM054-7, MBL); and goat anti-Ror1 for immunoprecipitation (AF2000, R&D Systems). Alexa Fluor–conjugated secondary antibodies, rhodamine- or Alexa Fluor 647–conjugated phalloidin and DQ-collagen IV were purchased from Thermo Fisher Scientific. Growth factor–reduced basement membrane matrix (Matrigel) and PLL were purchased from Corning and Sigma-Aldrich, respectively.

### Cell proliferation and survival assays

For cell proliferation assessment, the cells were transfected with the respective siRNAs through reverse transfection method and plated at a density of 1000 cells/well in a 96-well plate in triplicate with culture medium containing 10% (v/v) FBS. After the cells were cultured for 0, 1, 2, and 3 days, their viability was measured through the WST-8 assay using cell counting kit-8 (Dojindo) in accordance with the manufacturer’s instructions. For cell survival analysis, the cells were transfected with the respective siRNAs and cultured for 1 day in a 3.5 cm ϕ dish with culture medium containing 10% (v/v) FBS. The cells were then replated at a density of 1000 cells/well in a 96-well plate, precoated with either PLL (50 μg/ml) or undiluted Matrigel, with culture medium containing 1% (v/v) FBS. After the cells were cultured for 0 and 3 days, cell viability was measured using the WST-8 assay.

### Affinity precipitation, immunoprecipitation, and Western blotting

GST-mDia1-RBD and GST-rhotekin-RBD fusion proteins were expressed in *Escherichia coli* BL21(DE3) and purified using Glutathione-Sepharose. Active GTP-bound forms of Rif and RhoA were detected through affinity precipitation using 20 μg of GST-mDia1-RBD and GST-rhotekin-RBD, respectively, as reported previously (59). For immunoprecipitation and Western blotting, the cells were lysed in ice-cold lysis buffer (50 mM Tris–HCl, pH7.4, 0.5% (v/v) Nonidet P-40, 150 mM NaCl, 5 mM EDTA, 50 mM NaF, 1 mM Na_3_VO_4_, 1 mM *p*-PMSF, 10 μg/ml leupeptin, and 10 μg/ml aprotinin). To analyze phosphorylation of Dvl2 and Ror1, we cultured the cells overnight in dishes precoated with either PLL (50 μg/ml) or undiluted Matrigel. The cells were washed twice with PBS and lysed by incubating them in lysis buffer for 30 min at 4 °C without scraping. The resulting lysates were subjected to immunoprecipitation, SDS-PAGE, and immunoblotting as described previously (33). For the dephosphorylation of Dvl2 and Ror1, cell lysates were prepared and treated with calf intestinal alkaline phosphatase (Toyobo) as described previously (62). The ratio of phosphorylated and shifted Dvl2 (PS-Dvl2) to unshifted Dvl2 was measured as previously described (26). All uncropped Western blots can be found in the Supporting information.

### Cell surface biotinylation

The cells were washed three times with ice-cold PBS containing 0.9 mM CaCl_2_ and 0.49 mM MgCl_2_ (PBS+) and treated with 0.5 mg/ml Sulfo-NHS-ss-biotin (Thermo Fisher Scientific) in PBS+ for 30 min at 4 °C. The cells were then washed three times with Tris-quenching buffer (25 mM Tris-HC, pH7.5, 150 mM NaCl) at 4 °C to quench unreacted biotin prior to lysis in ice-cold lysis buffer (25 mM Tris–HCl, pH7.5, 150 mM NaCl, 5 mM EDTA, 1% (v/v) Triton X-100, 0.4% (w/v) sodium deoxycholate, 1 mM *p*-PMSF, 10 μg/ml leupeptin, and 10 μg/ml aprotinin). Biotin-labeled cell surface proteins in the lysate were collected on streptavidin–agarose beads and analyzed using Western blotting.

### Immunohistochemistry

Formalin-fixed, paraffin-embedded tissue microarray slides containing 50 cases of LUAD and matched adjacent or adjacent normal lung tissues were obtained from US Biomax Inc. (Cat No. LC1504, Rockville). Following deparaffinization and antigen retrieval with heated citrate buffer (pH 6.5), the tissue sections were permeabilized with 0.1% (v/v) Triton X-100 in PBS, and endogenous peroxides were blocked using hydrogen peroxide. Then, the sections were incubated with antibodies against Ror1 or Rif overnight at 4 °C and visualized using the Histofine Simple Stain MAX-PO (MULTI) kit (Nichirei). Nuclei were counterstained with hematoxylin. Among the 50 cases, 42 cases with papillary and/or tubular structures were analyzed for the expression of Ror1 and Rif.

### TCGA data analysis

*Rif* mRNA expression and clinical data of patients with LUAD were downloaded from TCGA using cBioPortal (63, 64) (http://www.cbioportal.org). Kaplan–Meier curves for overall survival based on *Rif* expression were generated using GraphPad Prism 9.0 (GraphPad Software Inc; https://www.graphpad.com) by setting the median expression of *Rif* as the cut-off. A log-rank test was conducted to assess the significance of the differences between survival curves.

### Cell staining on a 2D surface and matrigel

For imaging on a 2D surface, the cells were plated on glass coverslips (18 mm ø) precoated with fibronectin (10 μg/ml) or PLL (50 μg/ml) in a 12-well plate. The cells were transfected with the respective expression plasmids and cultured for 24 h before fixation with 4% (w/v) paraformaldehyde (PFA). The cells were permeabilized with 0.2% (v/v) Triton X-100 before phalloidin staining.

For imaging cells on Matrigel, 10 μl of undiluted Matrigel was placed on the center of glass coverslips (18 mm ø), spread by a pipet tip on ice, and then incubated at 37 °C for 30 min to solidify the Matrigel. Matrigel containing 25 μg/ml DQ-collagen IV was used to analyze ECM degradation. Cells either untransfected or transfected with the indicated expression plasmids or siRNAs were plated on coverslips in a 12-well plate and then cultured for 30 min, 1 h, or 2 h to assess Golgi polarity, filopodia formation, and ECM degradation, respectively. The samples were fixed with 4% (w/v) PFA containing 0.2% (w/v) glutaraldehyde, permeabilized with 0.2% (v/v) Triton X-100, and then stained with phalloidin and 4′,6-diamidino-2-phenylindole. For anti-GM130 immunostaining, the samples were fixed and treated with 5 mg/ml sodium borohydride. To quantify the number of filopodia, the samples were frozen in OCT compound (Sakura Finetek) and sectioned at a thickness of 20 μm perpendicular to the Matrigel surface in a cryostat before fixation with 4% (w/v) PFA. The fixed sections were permeabilized and stained with phalloidin and 4′,6-diamidino-2-phenylindole.

### Imaging analysis

Serial optical sections of the cells were obtained using LSM700 (Carl Zeiss) and A1 (Nikon) confocal microscopes and processed using ImageJ software (National Institutes of Health; https://imagej.nih.gov/ij/download.html). The intensity of the degraded DQ-collagen IV was quantified on z-stack images by measuring the fluorescence intensity of a cell divided by the background fluorescence intensity. To quantify the intensity of GM130, we stacked xz sections along the *y*-direction and defined the region of interest as a 120° sector emerging from the center of the nucleus toward the front and rear of the image. The fluorescence intensity of GM130 within the front and rear sectors were measured and expressed as a percentage of total GM130. The number of filopodia protruding from the area of the cell surface contacting Matrigel was counted.

### Transwell invasion assay

Transwell invasion assays were performed as described previously (60). In brief, Transwell inserts with a 10.5-mm diameter, 8-μm diameter pore size membrane (Corning) were coated with Matrigel (1:10 in serum-free RPMI1640). The lower surface of the membrane was coated with fibronectin (20 μg/ml in serum-free RPMI1640). Cells in serum-free RPMI1640 were loaded onto the upper well. The lower well was filled with RPMI1640 containing 10% (v/v) FBS. After incubation for 12 h, the cells were washed with PBS and fixed with 4% (w/v) PFA. The number of cells invading the lower surface of the membrane was counted.

### Generation of *Ror1*- , *Rif*-, or *Wnt5a*-KO PC9 cells

*Ror1*-, *Rif*-, *Wnt5a*-deficient PC9 cells were produced using the Alt-R CRISPR-Cas9 System (Integrated DNA Technologies ([IDT]) in accordance with the manufacturer’s protocol. Briefly, PC9 cells were transfected with an RNP complex composed of Cas9 nuclease V3 (IDT) and the following predesigned guide RNAs against the *Ror1*, *Rif*, or *Wnt5a* gene (IDT): *Ror1*#1 (Design ID: Hs.Cas9.ROR1.1. AA), *Ror1*#2 (Design ID: Hs.Cas9.ROR1.1. AC), *Rif*#1 (Design ID: Hs.Cas9.RHOF.1. AA), *Rif*#2 (Design ID: Hs.Cas9.RHOF.1. AB), *Wnt5a*#1 (Design ID: Hs.Cas9.WNT5A.1.AA), *Wnt5a*#2 (Design ID: Hs.Cas9.WNT5A.1.AB), or the negative control guide RNA #1 (IDT). After transfection, monoclonal PC9 cells were obtained through limiting dilution, and the successful knockout was confirmed by Western blotting.

### Xenograft assay

All animal experiments were approved by the Institutional Animal Care and Use Committee (permission number: P181003-R1) and carried out in accordance with the Kobe University Animal Experimentation Regulations. PC9 cells (Control, *Ror1* KO #1/#2, *Rif* KO #1/#2) at 2.5 × 10^5^ in 50% (v/v) Matrigel in PBS were subcutaneously transplanted into 6-week-old nude mice (BALB/cSlc-*nu/nu*) obtained from Japan SLC. At 28 days after transplantation, tumor tissues were isolated from mice euthanized under anesthesia and then weighed.

### RNA isolation and quantitative RT-PCR

Total RNAs were isolated using Sepasol-RNA I Super G (Nacalai Tesque) and reverse-transcribed using PrimeScript RT reagent kit (Takara). Real-time PCR was performed on the LightCycler 480 system (Roche Diagnostics) using LightCycler 480 SYBR Green I Master mix (Roche Diagnostics) or QuantStudio 6 Flex system (Thermo Fisher Scientific) using TB Green Premix Ex Taq II (Tli RNaseH Plus) (TaKaRa). The amount of mRNA was normalized relative to that of *18S rRNA*. The following primers were used: *Wnt5a*, 5′-TAAGCCCAGGAGTTGCTTTG-3′ (forward) and 5′-GCAGAGAGGCTGTGCTCCTA-3′ (reverse); *18S rRNA*, 5′-ATGGCCGTTCTTAGTTGGTG-3′ (forward) and 5′-CGCTGAGCCAGTCAGTGTAG-3′ (reverse).

### Statistical analysis

Significance was determined as ∗*p* < 0.05, ∗∗*p* < 0.01, or ∗∗∗*p* < 0.001 compared with the control using one-way ANOVA followed by Dunnett’s or Tukey’s post hoc test when more than three groups were analyzed or using Student’s *t* test when two groups were compared.

## Data availability

All data are contained within the article.

## Supporting information

This article contains supporting information.

## Conflict of interest

The authors declare that they have no conflicts of interest with the contents of this article.

## Supporting information

Supporting information

Uncropped blots(HHD)_R3
