# Supplementary material for: Rho family small GTPase Rif regulates Wnt5a-Ror1-Dvl2 signaling and promotes lung adenocarcinoma progression
Source: J Biol Chem. 2023 Sep 12;299(10):105248. doi: 10.1016/j.jbc.2023.105248 (PMC10570955; doi:10.1016/j.jbc.2023.105248)
Supplement: Supporting information [file mmc1.pdf]

Supporting information

Rho family small GTPase Rif regulates Wnt5a-Ror1-Dvl2 signaling and promotes lung adenocarcinoma progression

Michiru Nishita[1,#,*], Koki Kamizaki[2,#], Kyoka Hoshi[1], Kana Aruga[2], Ikumi Nishikaku[2], Hiroshi Shibuya[3], Kunio Matsumoto[4,5], Yasuhiro Minami[2,*]

[1]Department of Biochemistry, Fukushima Medical University School of Medicine, Fukushima, 960-1295, Japan
[2]Division of Cell Physiology, Department of Physiology and Cell Biology, Kobe University, Graduate School of Medicine, Kobe 650-0017, Japan
[3]Department of Molecular Cell Biology, Medical Research Institute, Tokyo Medical and Dental University (TMDU), Bunkyo-ku, Tokyo, 113-8510, Japan.
[4]Division of Tumor Dynamics and Regulation, Cancer Research Institute, Kanazawa University, Kakuma, Kanazawa 920-1192, Japan.
[5]WPI-Nano Life Science Institute, Kanazawa University, Kakuma, Kanazawa 920-1192, Japan.

[#]These authors contributed equally to this work.
[*]Corresponding authors.

Supplementary figure 1

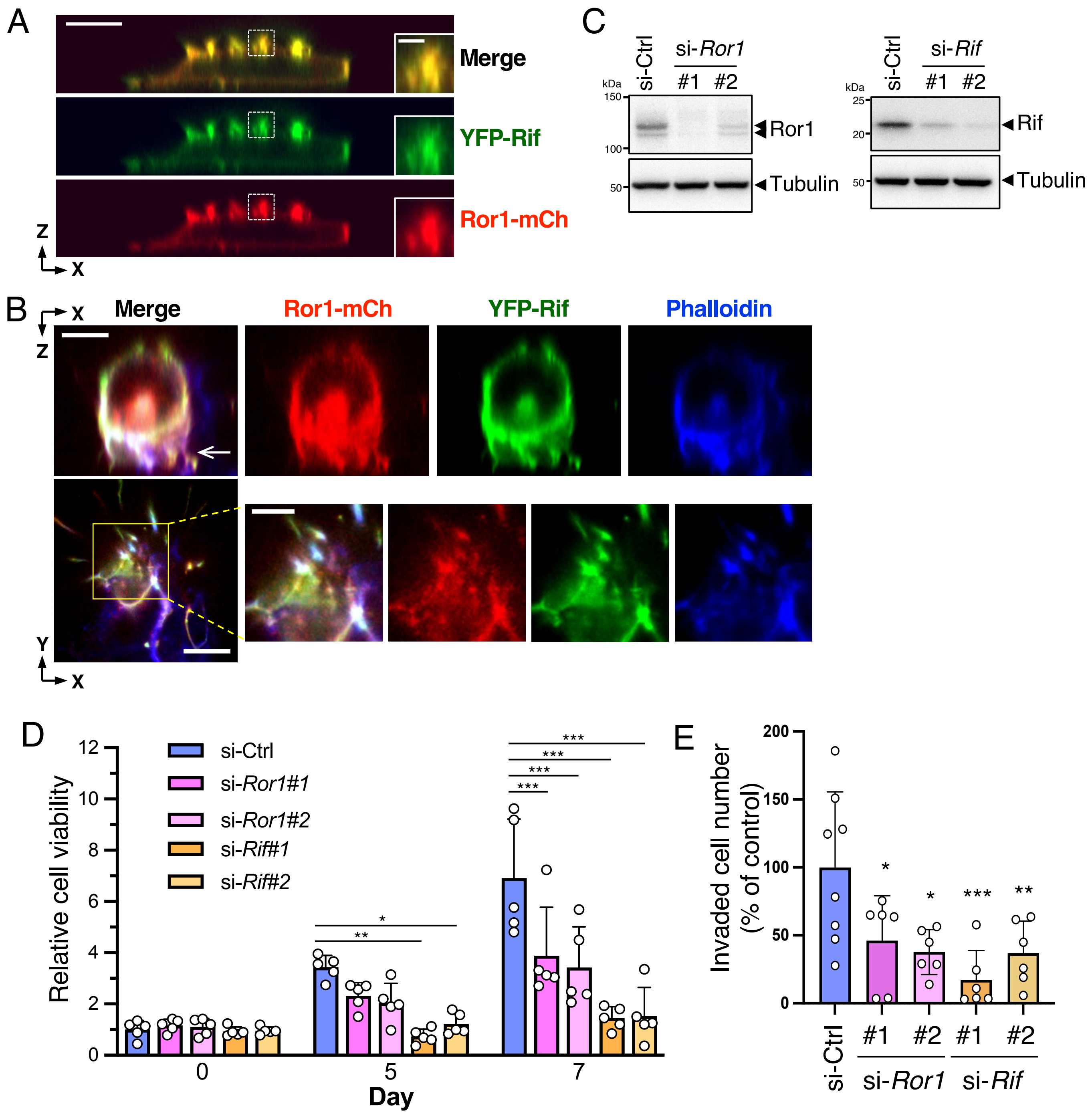

Figure S1. Ror1 and Rif are colocalized at filopodia and promote cell proliferation and invasion in HCC827 cells. Representative xz-images of cells expressing YFP-Rif and Ror1-mCh on a 2D surface (A) or Matrigel (B), showing colocalization of these proteins at filopodia. In (B), the xy images (lower panels) sectioned along the white arrow in xz confocal image (upper panels) are shown. Images are representative of two independent experiments. Scale bars, 10 µm. Magnified images of boxed regions are shown on the right. Scale bars in magnified images, 2 µm (A) and 5 µm (B). (C) Western blot analysis showing knockdown efficiency of siRNAs against *Ror1* and *Rif* in HCC827 cells. Blots are representative of two independent experiments. (D) Effects of *Ror1*- or *Rif*-knockdown on cell proliferation. Viability of cells transfected with the indicated siRNAs were assessed in media containing 10% FBS by using the WST-8 assay. Data are expressed as mean ± SD of five independent experiments performed in triplicate. (E) Transwell invasion assay showing decreased invasion of cells treated with si-*Ror1* or si-*Rif*. Data are expressed as mean ± SD of six to eight independent experiments. * $p < 0.05$, ** $p < 0.01$, *** $p < 0.001$, Tukey's test (D) and Dunnett's test (E).

Supplementary figure 2

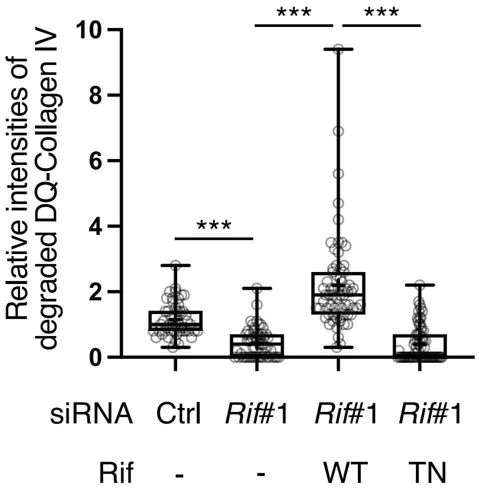

Figure S2. Reduced degradation of DQ-collagen IV in si-*Rif*#1-transfected cells can be reverted by the ectopic expression of wild-type Rif, but not its GDP-bound mutant. PC9 cells were transfected with si-*Rif*#1 and further infected with a recombinant retrovirus encoding Flag-tagged wild-type (WT) or GDP-bound mutant (TN) of Rif, which are resistant to si-*Rif*#1, or an empty vector (-), as indicated. Cells were cultured on Matrigel containing DQ-collagen IV for 2 h, and the intensity of the degraded DQ-collagen IV was quantified. Data are presented as a box-and-whisker plot. n = 50-70 cells from three independent experiments. *** $p < 0.001$, Tukey's test.

Supplementary figure 3

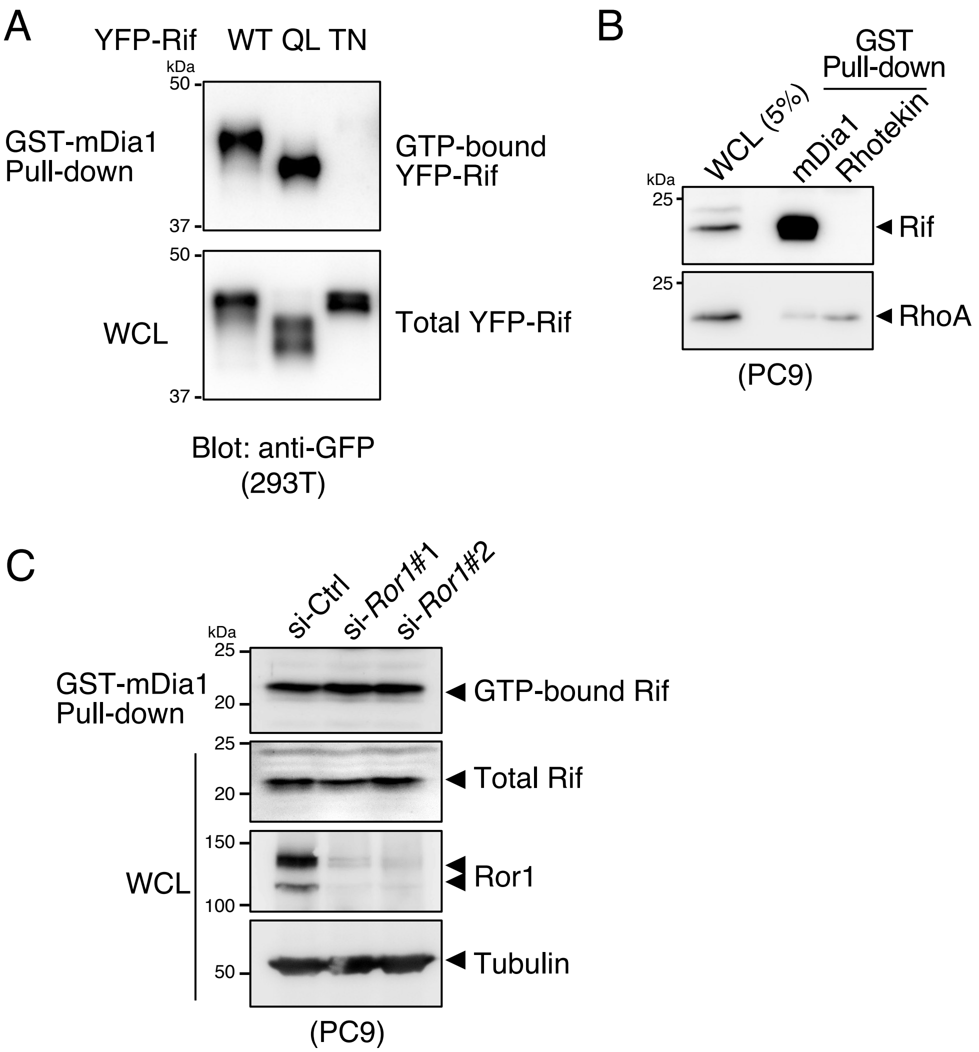

Figure S3. Rif exists as a GTP-bound active form irrespective of Ror1 expression in PC9 cells. (A) 293T cells were transfected with expression plasmids for wild-type (WT), GTP-bound form (QL), or GDP-bound form (TN) of YFP-Rif. Lysates were subjected to the effector pull-down assay by using GST-mDia1-RBD. Blots are representative of three independent experiments. (B) Lysates from PC9 cells were subjected to the effector pull-down assay by using GST-mDia1-RBD or GST-rhotekin-RBD. Blots are representative of two independent experiments. (C) Rif activity in PC9 cells transfected with siRNAs against *Ror1* were assessed by using the GST-mDia1-RBD pull-down assay. Blots are representative of four independent experiments.

Supplementary figure 4

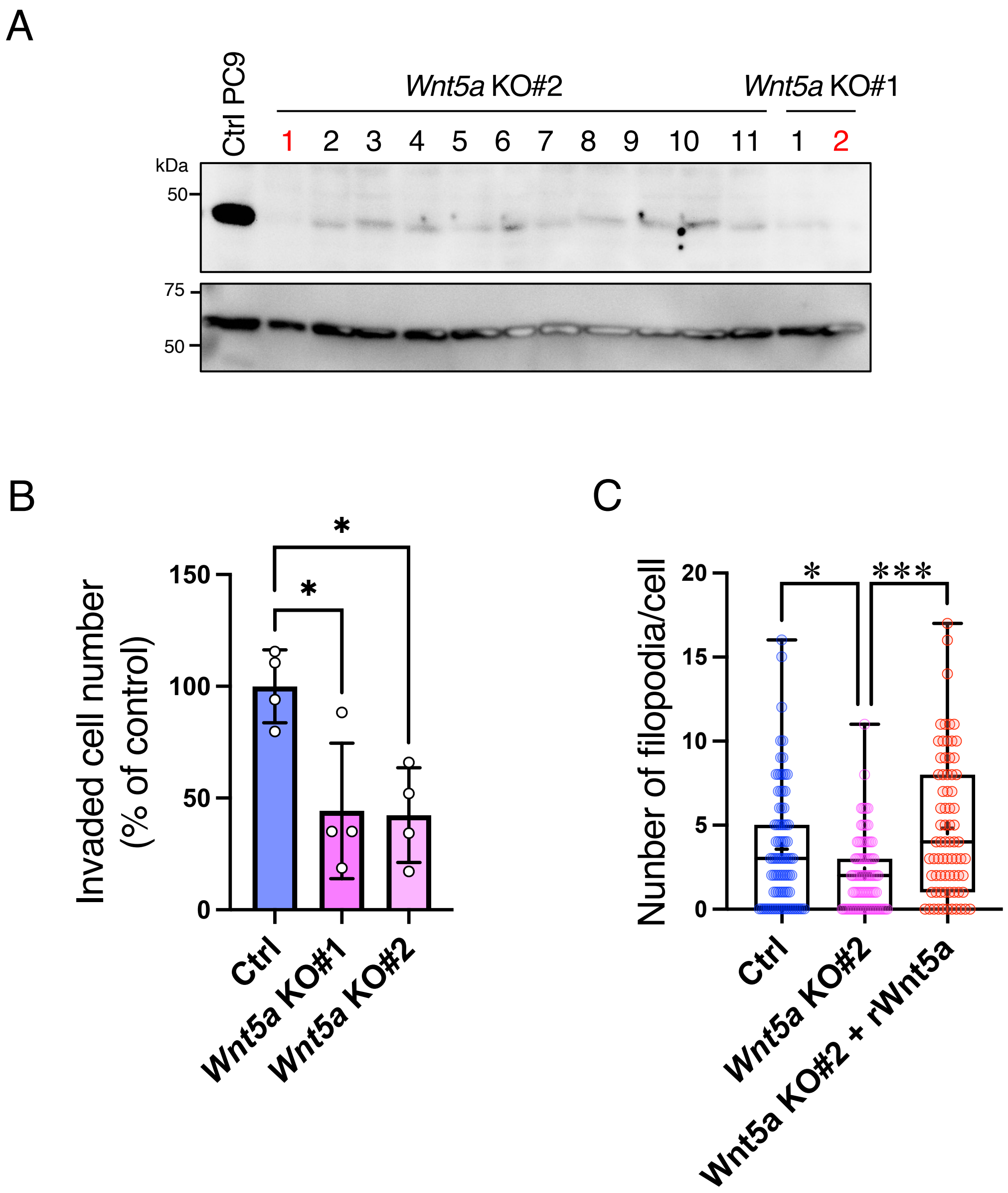

Figure S4. Autocrine Wnt5a production is required to promote invasion and filopodia formation, but not proliferation, in PC9 cells. (A) Representative western blots showing Wnt5a protein levels in whole cell lysates from control (ctrl) and *Wnt5a*-knockout (KO) PC9 clones. The *Wnt5a* KO clones generated with #1 and #2 gRNAs (lanes 14 and 2, indicated in red, respectively) were chosen for further analysis. (B) Transwell invasion assay showing decreased invasion in *Wnt5a*-KO PC9 cells compared to control (ctrl) PC9 cells. Data are expressed as mean ± SD of four independent experiments. * $p < 0.05$, Dunnett's test. (C) Ctrl or *Wnt5a*-KO#1 PC9 cells were cultured on Matrigel in the presence or absence of recombinant Wnt5a (rWnt5a; 200 ng/ml) for 1 h. Cells were stained with phalloidin and analyzed by confocal microscopy to quantify the number of filopodia/cell. Data are presented as a box-and-whisker plot. n = 73-94 cells from three independent experiments. * $p < 0.05$, *** $p < 0.001$, Tukey's test.

Supplementary figure 5

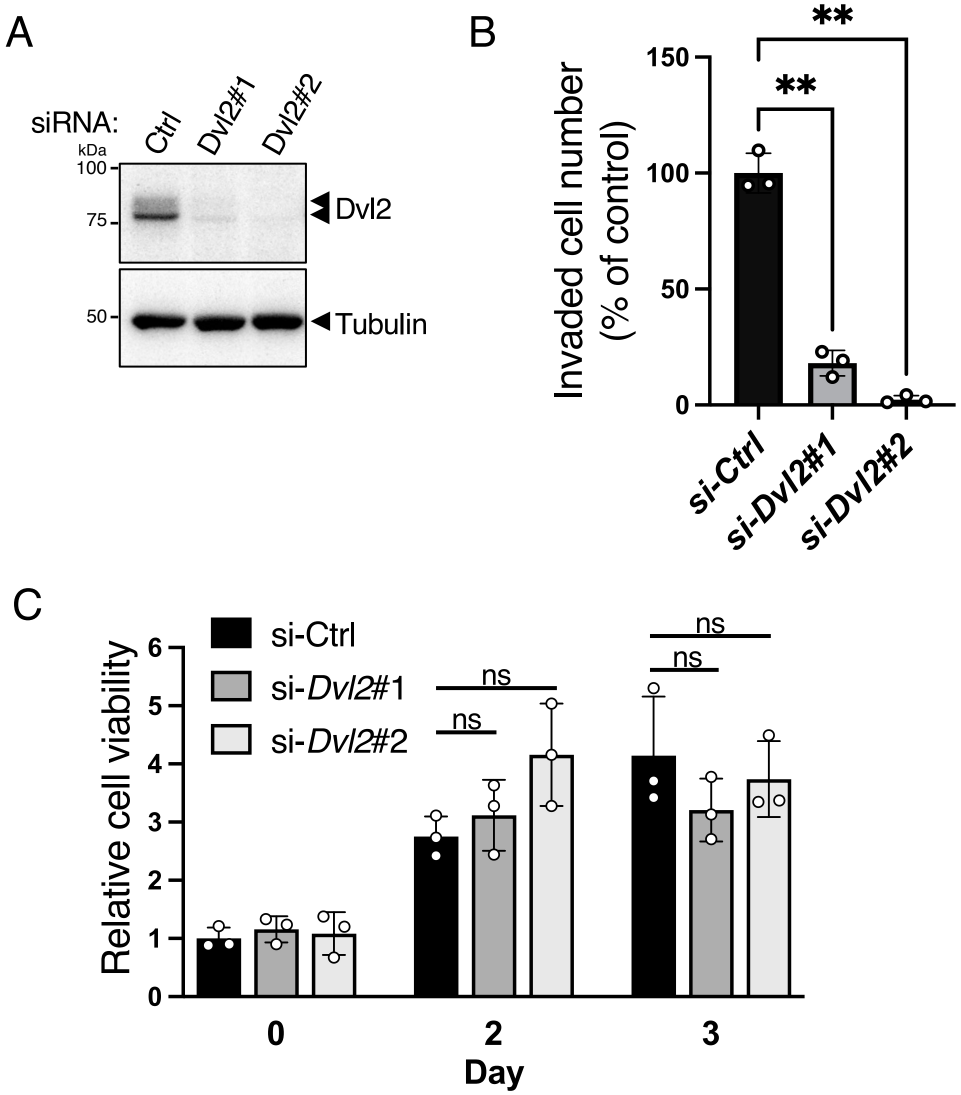

Figure S5. Dvl2 is required for invasive migration, but not proliferation, of PC9 cells. (A) Western blot analysis showing knockdown efficiency of siRNAs against *Dvl2* in PC9 cells. Images are representative of two independent experiments. (B) Transwell invasion assay showing decreased invasion of PC9 cells treated with si-*Dvl2*. Data are expressed as mean ± SD of three independent experiments. ** $p < 0.01$, Dunnett's test. (C) Effects of *Dvl2* knockdown on cell proliferation. Viability of cells transfected with the indicated siRNAs were assessed in media containing 10% FBS by using the WST-8 assay. Data are expressed as mean ± SD of three independent experiments. ns, not significant, Tukey's test.

Supplementary figure 6

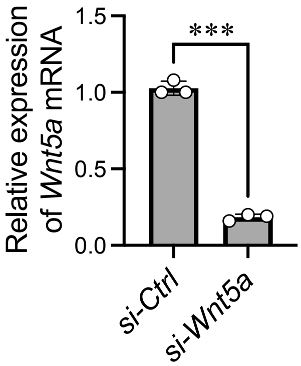

Figure S6. Quantitative RT-PCR analysis showing knockdown efficiency of si-*Wnt5a* in PC9 cells. Data are expressed as mean ± SD of three technical replicates of a representative experiment out of two independent experiments. ***P<0.001, t test.
