# Supplementary material for: Rho family small GTPase Rif regulates Wnt5a-Ror1-Dvl2 signaling and promotes lung adenocarcinoma progression
Source: J Biol Chem. 2023 Sep 12;299(10):105248. doi: 10.1016/j.jbc.2023.105248 (PMC10570955; doi:10.1016/j.jbc.2023.105248)

# Uncropped western blots

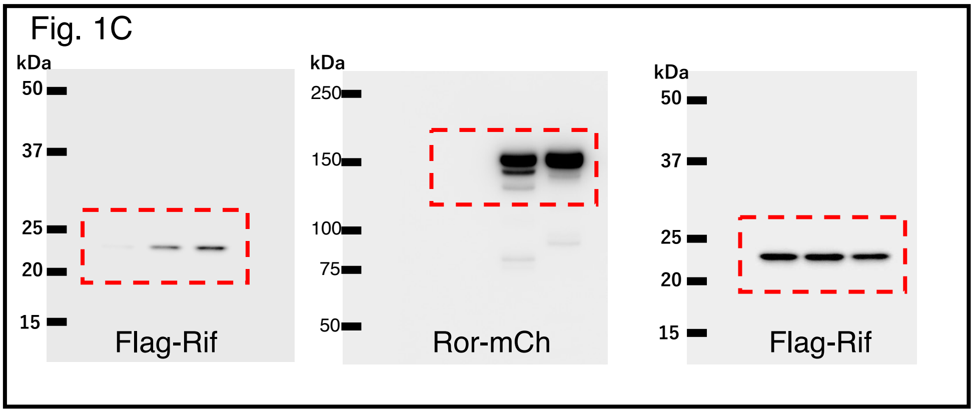


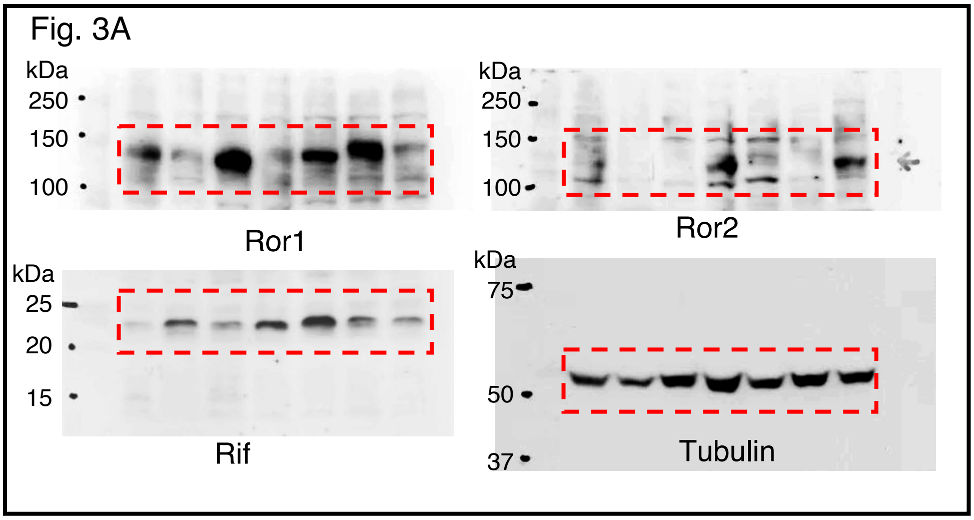


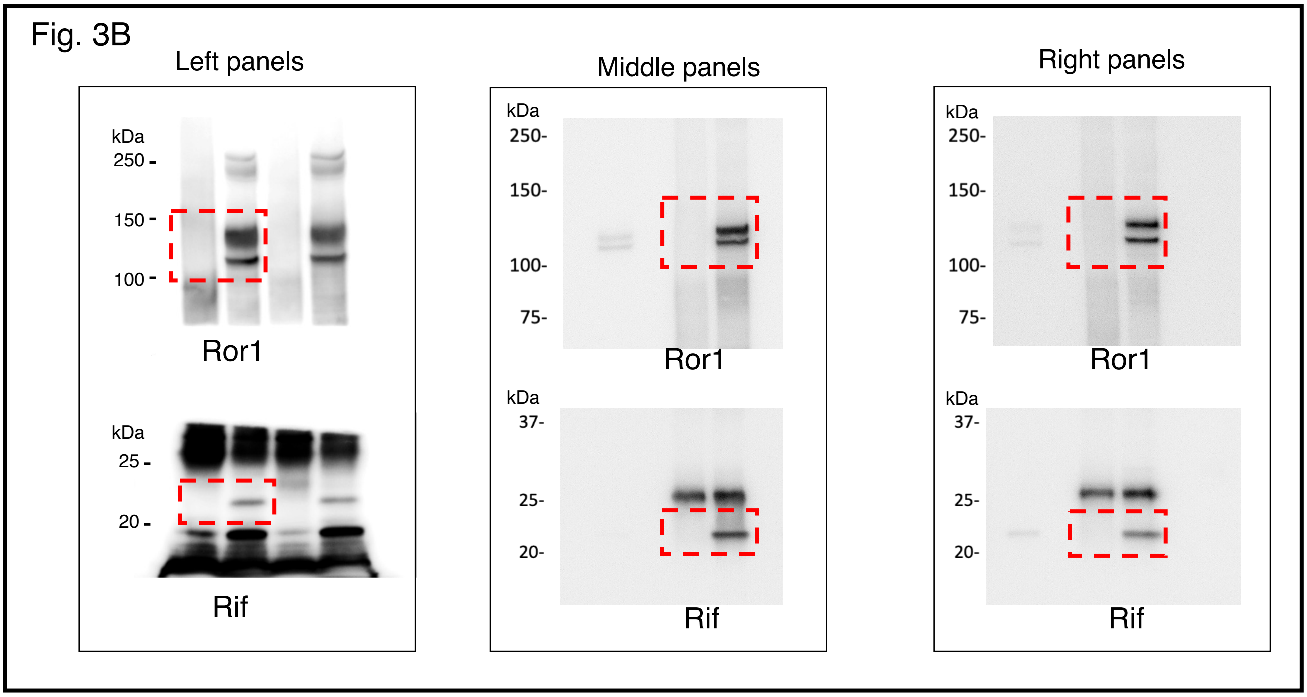

Fig. 4A

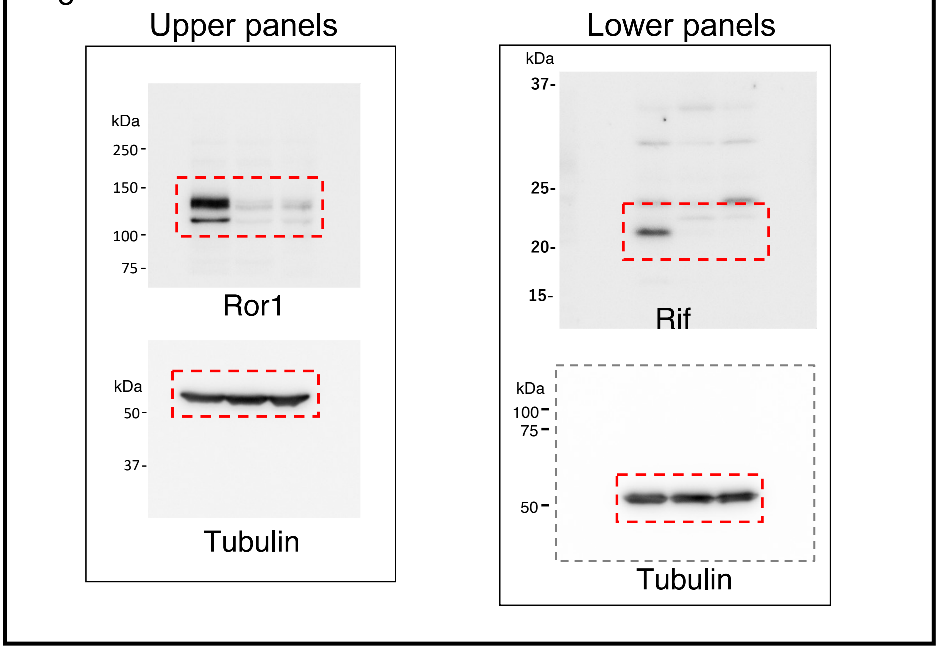

Upper panels
Lower panels
kDa
250
150
100
75
Ror1
kDa
50
37
Tubulin
kDa
37
25
20
15
Rif
kDa
100
75
50
Tubulin


Fig. 4E

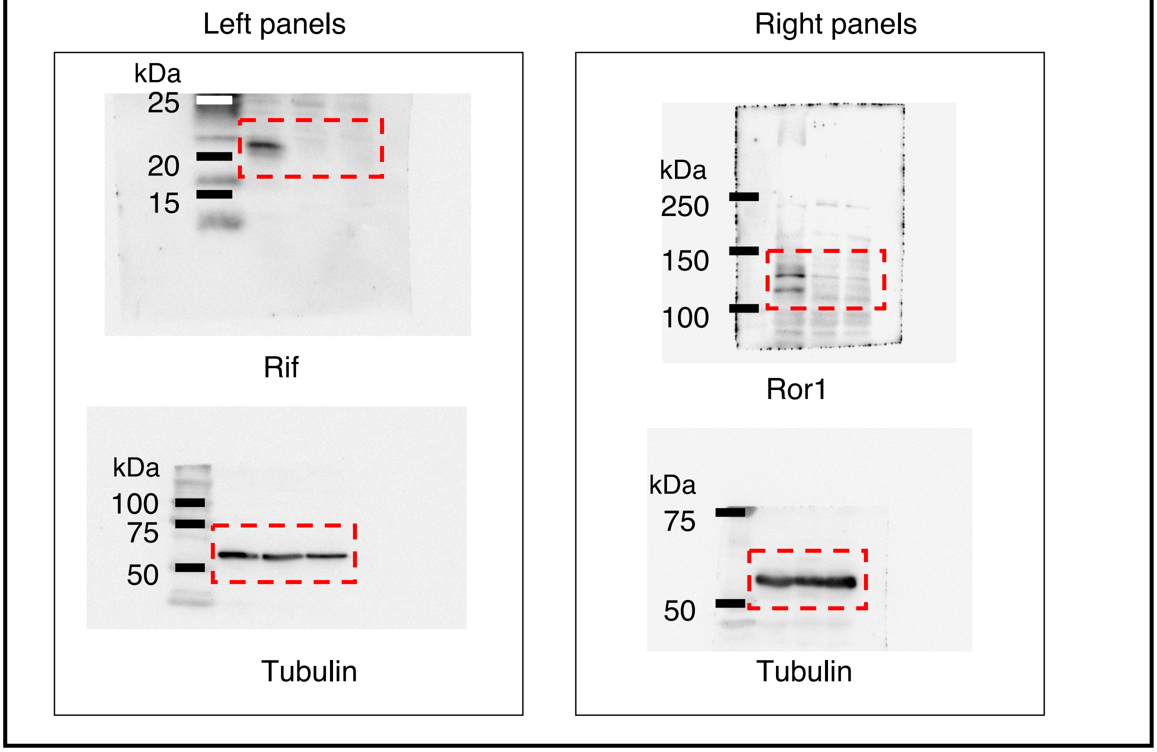

Left panels
Right panels
kDa
25
20
15
Rif
kDa
100
75
50
Tubulin
kDa
250
150
100
Ror1
kDa
75
50
Tubulin

Fig. 6A

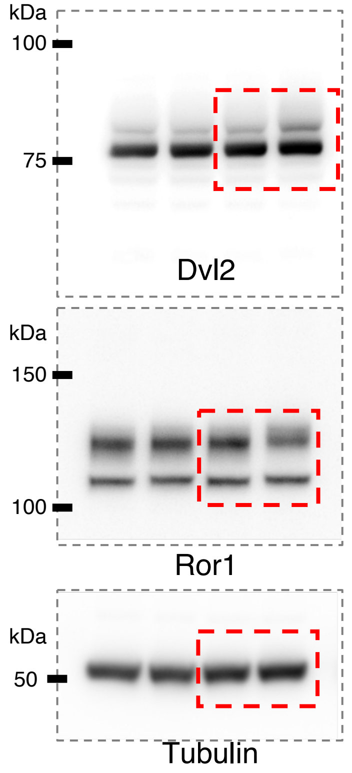


Fig. 6B

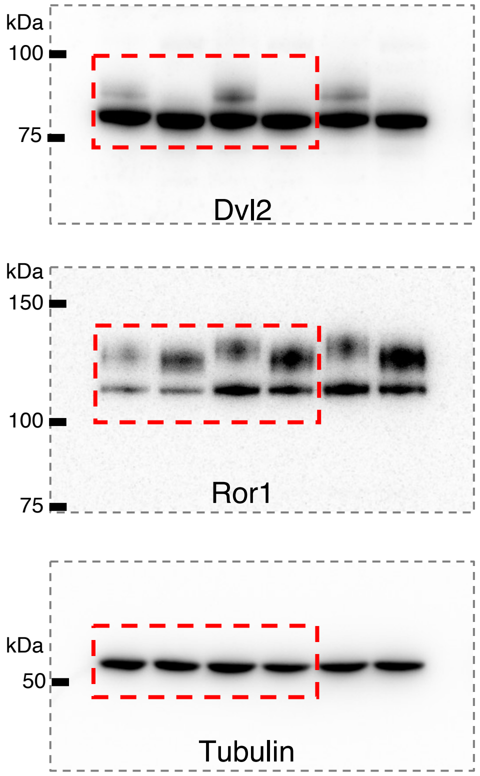


Fig. 6C

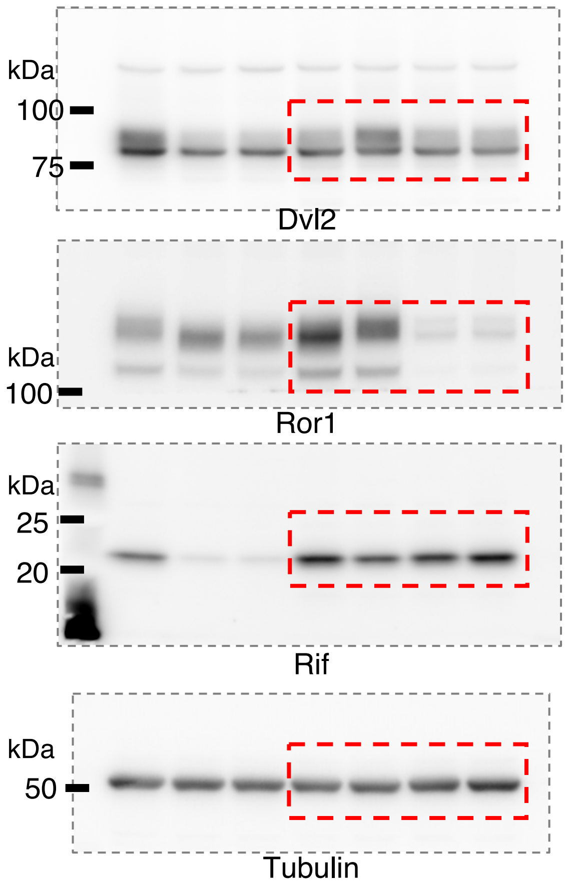


Fig. 6D

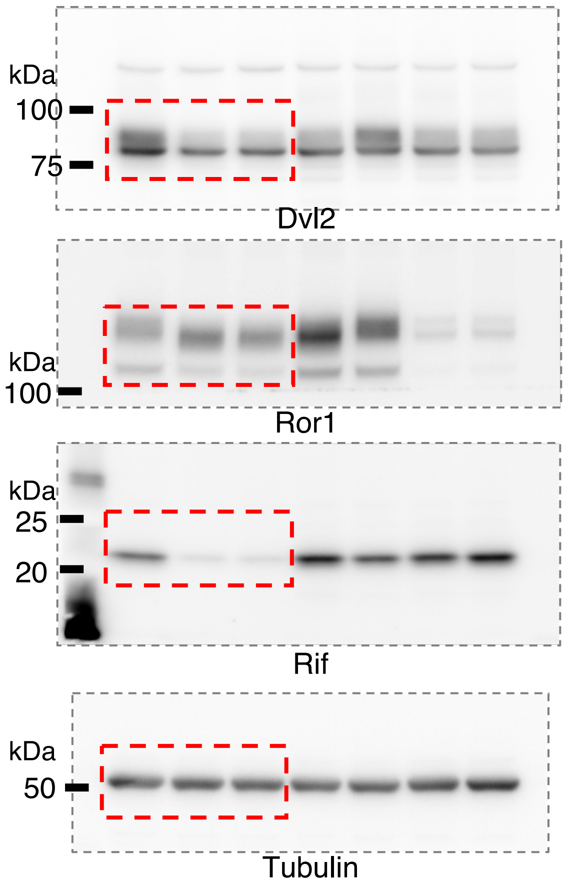

Fig. 6E

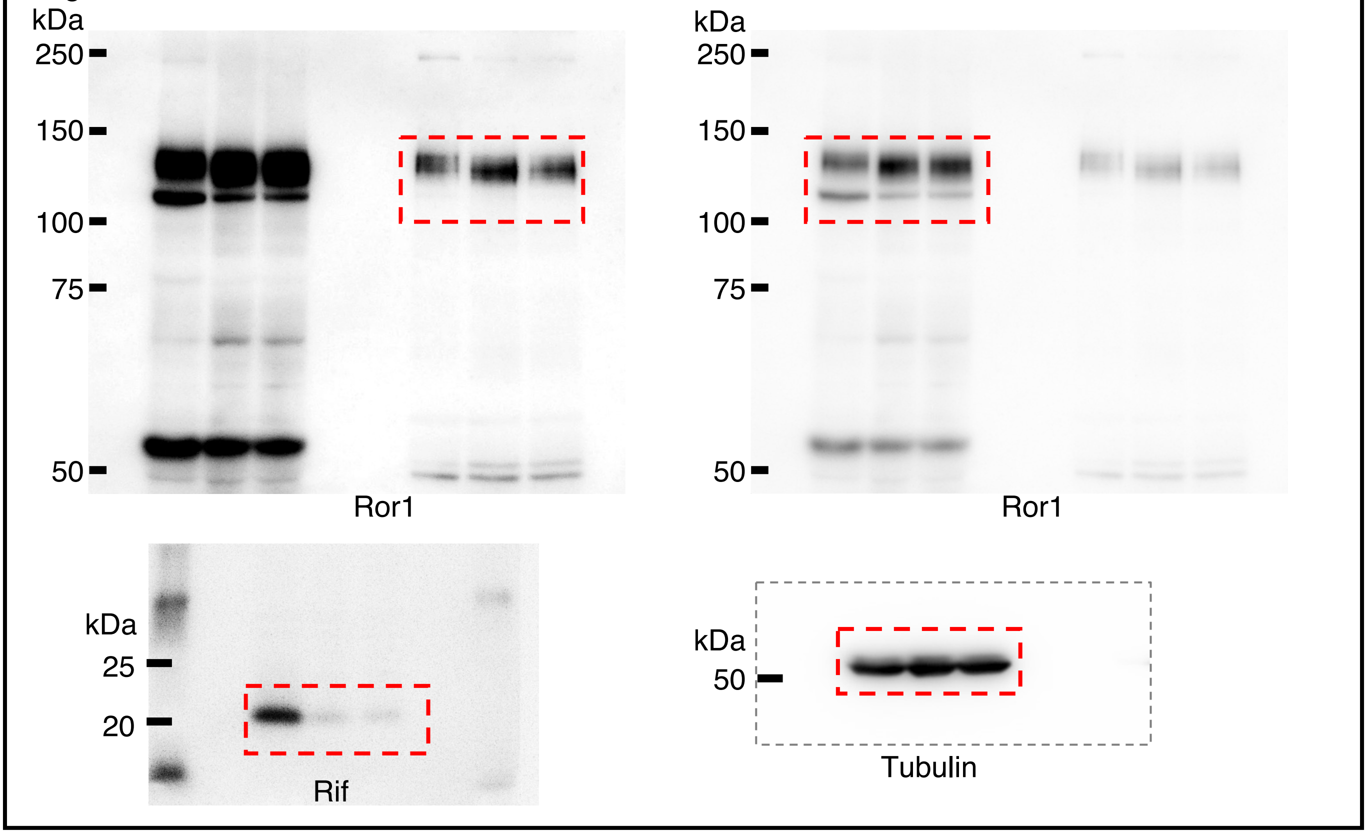


Fig. S1C

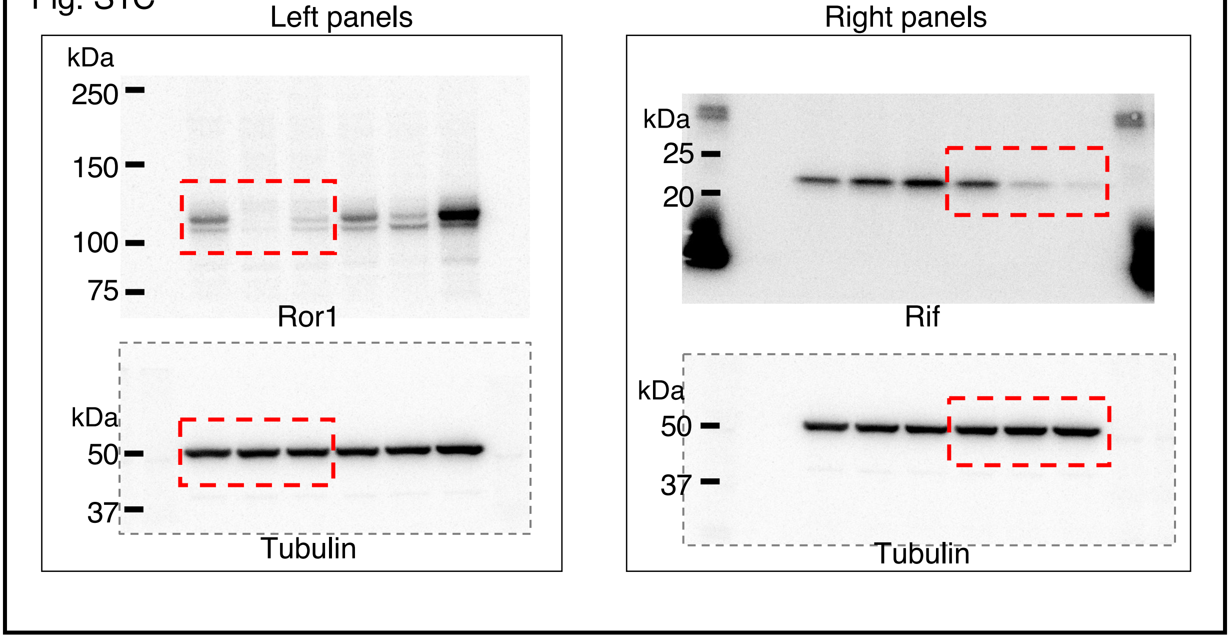

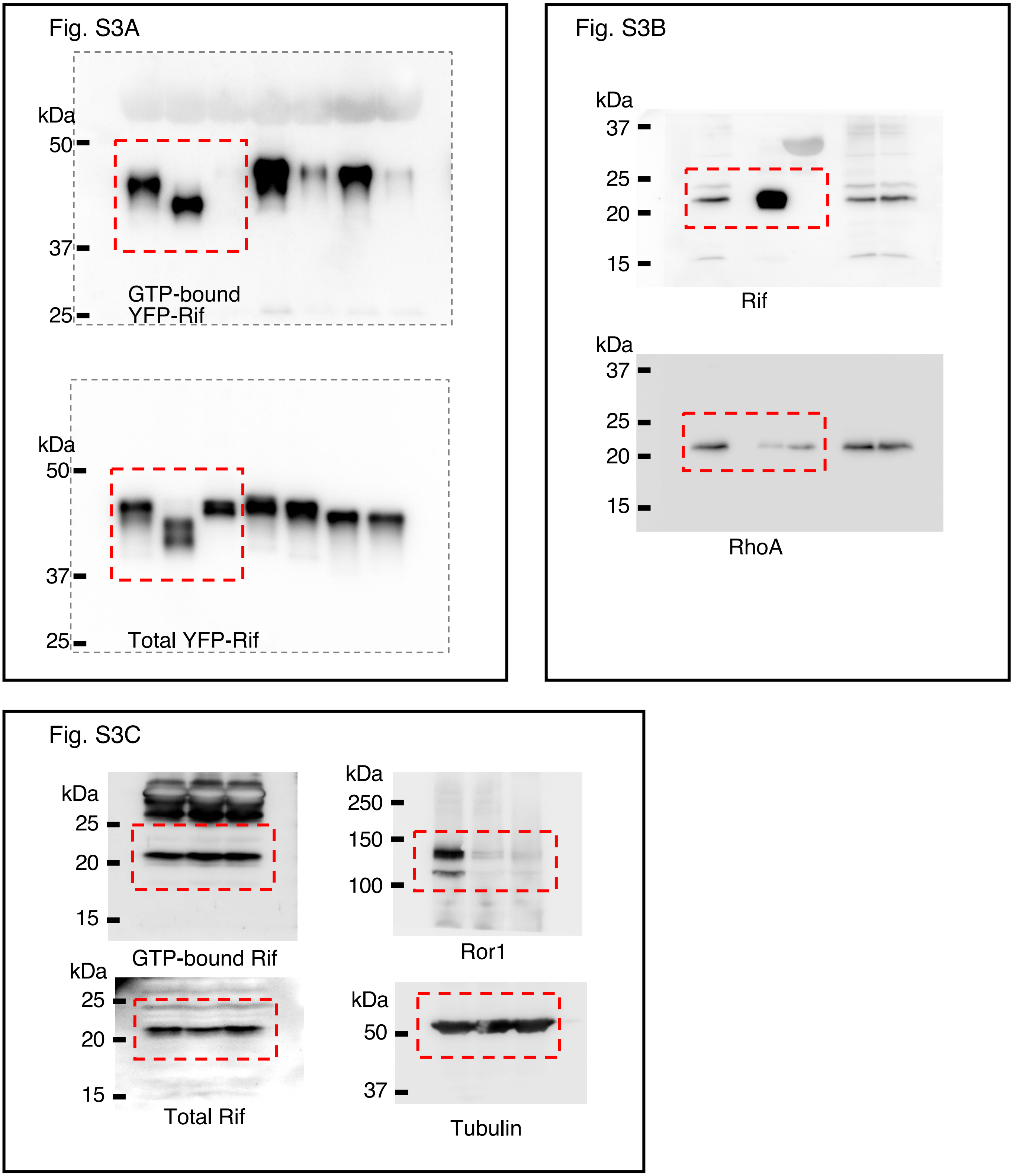

Fig. S3A
kDa
50
37
25
GTP-bound
YFP-Rif
kDa
50
37
25
Total YFP-Rif
Fig. S3B
kDa
37
25
20
15
Rif
kDa
37
25
20
15
RhoA
Fig. S3C
kDa
25
20
15
GTP-bound Rif
kDa
250
150
100
Ror1
kDa
25
20
15
Total Rif
kDa
50
37
Tubulin

Fig. S4A

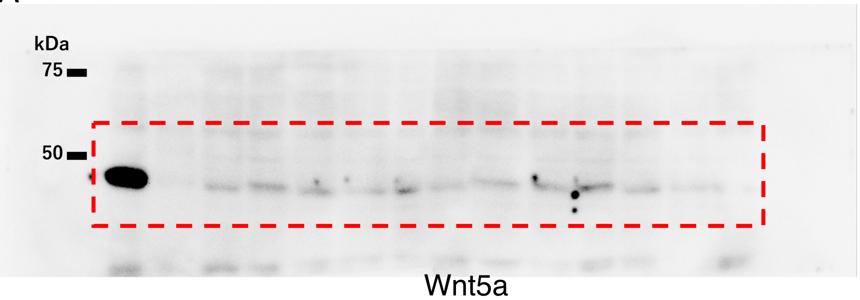


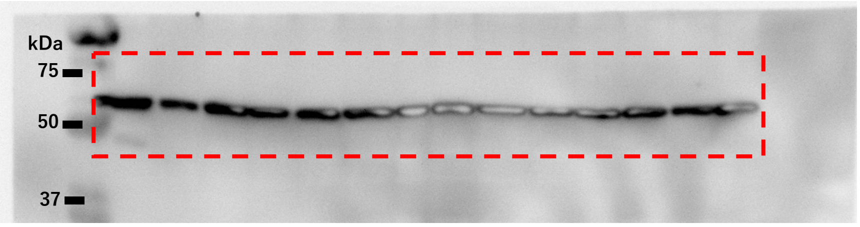


Fig. S5A

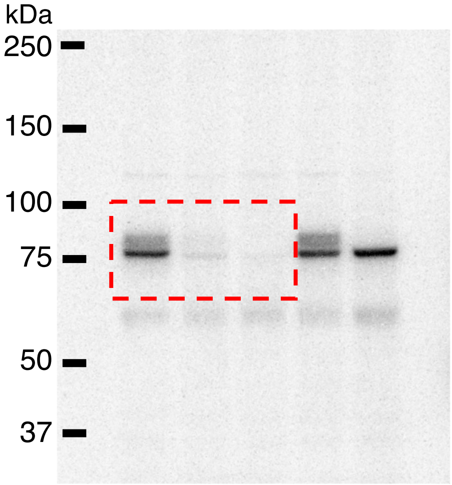


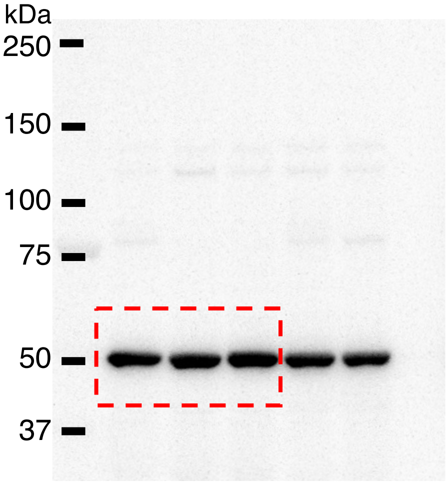

Supplement: Uncropped blots(HHD)_R3 [file mmc2.pdf]
